# Benchmark of *GW* Methods for Core-Level Binding Energies

**DOI:** 10.1021/acs.jctc.2c00617

**Published:** 2022-11-02

**Authors:** Jiachen Li, Ye Jin, Patrick Rinke, Weitao Yang, Dorothea Golze

**Affiliations:** † Department of Chemistry, Duke University, Durham, North Carolina27708, United States; ‡ Department of Applied Physics, Aalto University, Otakaari 1, FI-02150Espoo, Finland; § Faculty of Chemistry and Food Chemistry, Technische Universität Dresden, 01062Dresden, Germany

## Abstract

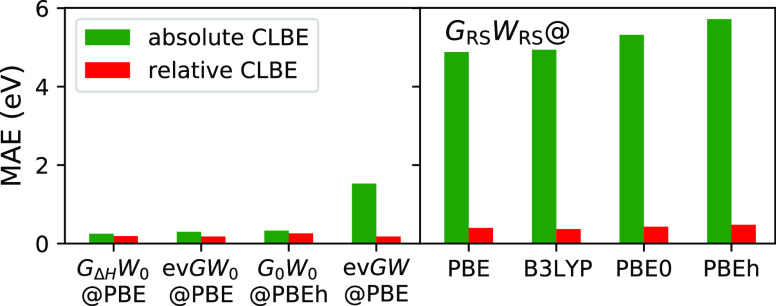

The *GW* approximation has recently gained increasing attention as a viable method for the computation of deep core-level binding energies as measured by X-ray photoelectron spectroscopy. We present a comprehensive benchmark study of different *GW* methodologies (starting point optimized, partial and full eigenvalue-self-consistent, Hedin shift, and renormalized singles) for molecular inner-shell excitations. We demonstrate that all methods yield a unique solution and apply them to the CORE65 benchmark set and ethyl trifluoroacetate. Three *GW* schemes clearly outperform the other methods for absolute core-level energies with a mean absolute error of 0.3 eV with respect to experiment. These are partial eigenvalue self-consistency, in which the eigenvalues are only updated in the Green’s function, single-shot *GW* calculations based on an optimized hybrid functional starting point, and a Hedin shift in the Green’s function. While all methods reproduce the experimental relative binding energies well, the eigenvalue self-consistent schemes and the Hedin shift yield with mean absolute errors <0.2 eV the best results.

## 1 Introduction

X-ray photoelectron spectroscopy (XPS) is a standard characterization tool for materials,^1^ liquids,^2,3^ and molecules.^4^ In XPS, the core-level binding energies (CLBEs) are measured, which are element-specific but also sensitive to the chemical environment.^5^ However, establishing the link between the measured spectrum and the atomic structure is challenging, in particular for complex materials with heavily convoluted XPS signals.^6,7^ Guidance from theory is thus often necessary to interpret XPS spectra.

Theoretical XPS methods can be distinguished into delta and response approaches. In delta-based methods, the CLBE is computed as the total energy difference between the neutral and core-excited system. These calculations can be performed at different levels of theory, for example, with high-level wave function methods, such as delta coupled cluster (ΔCC)^8,9^ or with Kohn–Sham density functional theory^10−12^ (KS-DFT). The most popular DFT-based approach is the delta self-consistent field (ΔSCF) method,^13^ which has been thoroughly benchmarked.^14−20^ While high accuracy can be achieved with these approaches, the explicit optimization of a core-ionized wave function leads to conceptual problems, for example, regarding periodicity, constraining spin–orbit coupled states or, in the case of DFT, deteriorating accuracy for larger structures, which was already discussed and demonstrated elsewhere.^21−25^

An explicit orbital optimization of core-ionized systems and the related conceptual issues are avoided in response theories, where electron propagators are applied to transform the ground into an excited state. Recently, wave function-based methods, such as linear response and equation-of-motion CC methods^26−29^ and the algebraic diagrammatic construction method,^28^ were reassessed for absolute CLBEs, yielding partly promising results. Another promising approach in the realm of response methods is the *GW* approximation^30−32^ to many-body perturbation theory, which is derived from Hedin’s equation^33^ by omitting the vertex correction. The *GW* approximation is considered the “gold standard” for the computation of band structures of materials,^31,34^ but it has also been successfully applied to valence excitations of molecules.^31,35−37^

Due to its primary application to solids, *GW* was traditionally implemented in plane wave codes that typically use pseudo-potentials for the deeper states. With the increasing availability of the *GW* method in localized basis set codes,^38−45^ core states moved into focus. CLBE calculations have emerged as a recent trend in *GW*.^22,24,44−53^ By extension to the Bethe–Salpeter equation (BSE@*GW*), also K-edge transition energies measured in X-ray absorption spectroscopy can be calculated.^54^ These studies focused primarily on molecules. However, *GW* is one of the most promising methods for core-level predictions of materials because the scaling with respect to system size is generally smaller than for wave function-based response methods and the method is well established for periodic structures. In addition, *GW* implementations for localized basis sets are advancing rapidly. Recently, periodic implementations^44,55,56^ and low-scaling *GW* algorithms with  complexity emerged in localized basis set formulations.^43,57−60^

The application of *GW* to core states is more difficult than for valence states. We showed that more accurate and computationally more expensive techniques for the frequency integration of the self-energy are required compared to valence excitations.^22^ Furthermore, we found that the standard single-shot *G*_0_*W*_0_ approach performed on top of DFT calculations with generalized gradient approximations (GGAs) or standard hybrid functionals fails to yield a distinct solution, which is caused by a loss of spectral weight in the quasiparticle (QP) peak.^49^ We demonstrated that eigenvalue self-consistency in *G* or using a hybrid functional with 45% exact exchange as the starting point for the *G*_0_*W*_0_ calculation restores the QP main excitation. Including also relativistic corrections,^50^ an agreement of 0.3 and 0.2 eV with respect to experiment was reported for absolute and relative CLBEs, respectively.^49^

While *G*_0_*W*_0_ is the computationally least expensive *GW* flavor, it strongly depends on the density functional approximation (DFA). Tuning the exchange in the hybrid functional to, for example, 45% is conceptually unappealing and introduces undesired small species dependencies, as discussed more in detail in this work. Self-consistency reduces or removes the dependence on the underlying DFT functional but significantly increases the computational cost. The computationally least expensive self-consistent schemes are the so-called eigenvalue self-consistent approaches, where the eigenvalues are iterated in the Green’s function *G* (ev*GW*_0_) or alternatively in *G* and the screened Coulomb interaction *W* (ev*GW*).^31^ Higher-level self-consistency schemes, such as fully self-consistent *GW*(61−63) (sc*GW*) and QP self-consistent *GW*(64) (qs*GW*), remove, unlike ev*GW*_0_ or ev*GW*, the starting point dependence completely. However, these higher-level self-consistency schemes are much more expensive and not necessarily better because of the inherent underscreening due to the missing vertex correction.^65,66^

Recently, we proposed the renormalized singles (RS) Green’s function approach, denoted as *G*_RS_*W*_0_, to reduce the starting point dependence in *GW*.^67^ The RS concept was developed in the context of the random phase approximation (RPA) for accurate correlation energies^68,69^ and termed renormalized single excitation (RSE) correction. Following standard perturbation theory, SEs contribute to the second-order correlation energy. It was shown that their inclusion significantly improves binding energies (BEs).^68,69^ The RS Green’s function approach extends the RSE idea from correlation energies to *GW* QP energies. In the *G*_RS_*W*_0_ scheme, the RS Green’s function is used as a new starting point and the screened Coulomb interaction is calculated with the KS Green’s function. For valence excitations, we found that this renormalization process significantly reduces the starting point dependence and provides improved accuracy over *G*_0_*W*_0_.^67^ The mean absolute errors (MAEs) obtained from the *G*_RS_*W*_0_ approach with different DFAs are smaller than 0.2 eV for predicting ionization potentials of molecules in the *GW*100 set.^67^ Unlike the self-consistent schemes, the RS Green’s function method hardly increases the computational cost compared to *G*_0_*W*_0_.

Recently, we employed the concept of RS in a multireference DFT approach for strongly correlated systems.^70^ We also used the RS Green’s function in the *T*-matrix approximation (*G*_RS_*T*_RS_).^71^ The *T*-matrix method scales formally as  with respect to system size *N*,^71,72^ with reduced scaling possible using effective truncation of the active space.^73^ In addition to the high computational cost, the performance of *G*_RS_*T*_RS_ for core levels is not particularly impressive. The error with respect to experiment is 1.5 eV for absolute and 0.3 eV for relative CLBEs.^71^ In the present work, we focus thus on RS *GW* approaches for core levels.

In this work, we benchmark *GW* approaches, which we consider computationally affordable and suitable for large-scale applications. This includes *G*_0_*W*_0_ with tuned starting points, the eigenvalue self-consistent schemes ev*GW*_0_ and ev*GW*, and two new methods that we introduce in this work. One is based on the so-called Hedin shift^74^ and can be understood as approximation of the ev*GW*_0_ method. We refer to this scheme as *G*_Δ*H*_*W*_0_. The other is a different flavor of the RS Green’s function approach, where the screened Coulomb interaction is also computed with the RS Green’s function (*G*_RS_*W*_RS_).

The remainder of this article is organized as follows: we introduce the different *GW* approaches in Section 2 and give the computational details for our calculations in Section 3. The solution behavior of the different methods is discussed in Section 4.1 by comparing self-energy matrix elements and spectral functions. In Section 4.2, results are presented for the CORE65 benchmark set and in Section 4.3 for the ethyl trifluoroacetate (ETFA) molecule. The computational cost for the different *GW* flavors is discussed in Section 4.4, and we finally draw conclusions in Section 5.

## 2 Theory

### 2.1 Single-Shot *G*_0_*W*_0_ Approach

The most popular *GW* approach is the single-shot *G*_0_*W*_0_ scheme, where the *GW* QP energies are obtained as corrections to the KS eigenvalues {ϵ_*n*_^0^}
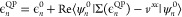
1where {ψ_*n*_^0^} are the KS molecular orbitals (MOs) and *v*^*xc*^ is the KS exchange–correlation potential. We use *i*, *j* for occupied orbitals, *a*, *b* for virtual orbitals, and *m*, *n* for general orbitals. We omitted the spin index in all equations for simplicity and use the notation  and  in the following. We can directly obtain the CLBE of state *n* from the QP energies because they are related by CLBE_*n*_ = −ϵ_*n*_^QP^. The self-energy Σ is given by
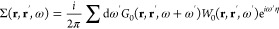
2where the noninteracting KS Green’s function is denoted *G*_0_ and the screened Coulomb interaction *W*_0_. η is a positive infinitesimal. The KS Green’s function reads
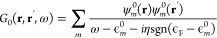
3where ϵ_F_ is the Fermi energy. The screened Coulomb interaction is calculated at the level of the RPA as
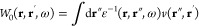
4where ε(**r**,**r**′,ω) is the dielectric function and *v*(**r**,**r**′) = 1/|**r** – **r**′| the bare Coulomb interaction.

The calculation of the self-energy matrix elements Σ_*n*_ is split into a correlation part Σ^*c*^ and an exchange part Σ^*x*^, that is, Σ_*n*_ = Σ_*n*_^*c*^ + Σ_*n*_^*x*^. The HF-like exchange part Σ_*n*_^*x*^ is given by
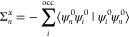
5The correlation part Σ^*c*^ is computed from *W*_0_^*c*^ = *W*_0_ – *v* and is the part that we plot in Section 4.1 to investigate the *GW* solution behavior. The correlation part in its fully analytic form is given by
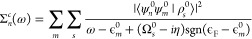
6where Ω_*s*_^0^ are charge neutral excitations at the RPA level and ρ_*s*_^0^ are the corresponding transition densities. The fully analytic form of Σ_*n*_^*c*^ directly shows the pole structure of the self-energy and is illustrative to understand the solution behavior of *GW*. However, the evaluation of Ω_*s*_^0^ scales with . In practice, the correlation self-energy is usually evaluated with a reduced scaling by using techniques such as analytical continuation or the contour deformation; see ref (31) for an overview of frequency integration techniques.

The QP energies can be obtained by solving eq 1, which is typically the computationally least expensive approach. In this work, we additionally employ two alternative approaches to obtain further insight into the physics and suitability of the different *GW* approaches. The first is the graphical solution of eq 1, where we plot the self-energy matrix elements Σ_*n*_^*c*^ and determine the QP solution by finding the intersections with the straight line ω – ϵ_*n*_^0^ + *v*_*n*_^*xc*^ – Σ_*n*_^*x*^. The presence of several intersections would indicate that more than one solution exists. The second, computationally even more expensive alternative, is the computation of the spectral function,^49^ which is given by
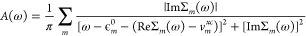
7The spectral function is related to the photocurrent, which is the experimental observable in photoemission spectroscopy, as discussed in, for example, refs (31) and (75). In eq 7, we include also the imaginary part of the complex self-energy, which gives us direct access to the spectral weights and satellite spectrum.

The *GW* formalism is primarily applied to closed-shell systems, but the treatment of open shell systems is also possible to some extent. Open shell systems are often multireference problems, while the KS Green’s function *G*_0_ assumes a nondegenerate ground state characterized by a single Slater determinant. If a particular ground state is a sum of several Slater determinants, the *GW* approach is not applicable and a multiconfiguration method should be used instead. If it is possible to choose among the multiple ground states of the open shell systems one that evolves into a single Slater determinant, for example, the triplet state of O_2_, then our definition of *G*_0_ and thus Dyson’s equation are valid. As shown in ref (76), the QP multiplet splittings of valence excitations are correctly predicted by *GW* if the preceding spin-polarized KS-DFT calculations provide a sufficient approximation of the particular ground states of the open shell system. While the multiplet splittings of open shell valence levels can be rather complex, the 1s excitations give rise to a simple doublet with a weaker line at higher BE and a stronger one at lower BE.^4^ So far, we only investigated the O_2_ molecules and found that *GW* performed very well for the spitting of the O 1s lines.^49^

### 2.2 Eigenvalue Self-Consistent *GW* Schemes

Including self-consistency in Hedin’s *GW* equations is a widely used strategy to go beyond *G*_0_*W*_0_. sc*GW*^31,63^ is conceptually the purest approach but also the most expensive one. To reduce the computational demands, different lower-level self-consistent schemes were developed. The simplest approach is an eigenvalue self-consistent scheme, which comes in two different flavors. The first one is to iterate the eigenvalues only in *G* and keep *W* fixed at the *W*_0_ level. This scheme is referred to as the ev*GW*_0_ approach. In ev*GW*_0_, we start by updating the KS eigenvalues in the Green’s function with the *G*_0_*W*_0_ QP energies, re-evaluate the QP equation (see eq 1), and iterate until self-consistency in *G* is reached. The Green’s function in the eigenvalue self-consistent scheme reads
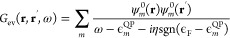
8with ϵ_*m*_^QP^ = ϵ_*m*_^0^ + Δϵ_*m*_, where Δϵ_*m*_ is the *GW* correction, see eq 1. The second flavor is ev*GW*, where the KS eigenvalues are updated not only in *G* but also in the screened Coulomb interaction *W*. The eigenvalue self-consistent calculations are computationally significantly more expensive than a *G*_0_*W*_0_ calculation, in particular in combination with the accurate self-energy integration techniques that are required for core levels.^22^ The computational demands are large because *G*, the screened Coulomb interaction *W* (in the case of ev*GW*), and the self-energy have to be built repeatedly. In addition, eq 1 must be solved not only for the states of interest but for all states.

### 2.3 *GW* with the Hedin Shift

The cost of an ev*GW*_0_ scheme can be drastically reduced by using a global shift Δ*H* instead of an individual shift Δϵ_*m*_ for each state *m*. This scheme was first introduced by Hedin^33^ and is referred to as *G*_Δ*H*_*W*_0_ in the following. The *G*_Δ*H*_*W*_0_ approach was discussed several times in the literature,^74,77−79^ and the effect of ev*GW*_0_ and *G*_Δ*H*_*W*_0_ on the self-energy structure has been discussed for valence states in ref (31). In the Hedin-shift scheme, the Green’s function transforms into
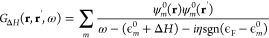
9where *G*_0_(ω – Δ*H*) = *G*_Δ*H*_(ω). The QP equation with the Hedin-shift scheme then becomes
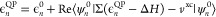
10

Traditionally, Δ*H* is determined with respect to the Fermi level of *G*_0_ for metals or the valence band maximum for gapped solid-state systems. For the molecular case, Δ*H* is evaluated with respect to the highest occupied MO (HOMO) by introducing the self-consistency condition ϵ_HOMO_^QP^ = ϵ_HOMO_^0^ + Δ*H*, which is inserted in eq 10 and yields
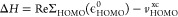
11As demonstrated in ref (31), ev*GW*_0_ and *G*_Δ*H*_*W*_0_ lead to a shift of the pole structure of the self-energy matrix elements Σ_*n*_ to more negative frequencies. For Σ_HOMO_(ω), the shift is similar for ev*GW*_0_ and *G*_Δ*H*_*W*_0_, yielding practically the same ϵ_HOMO_^QP^.

For core states, we found that the shift Δ*H* computed as in eq 11 is much smaller than the one from ev*GW*_0_. Taking the H_2_O molecule as an example, the ev*GW*_0_ shift of the self-energy poles with respect to *G*_0_*W*_0_ is in the range of −6 eV for the HOMO, whereas it is in the range of −30 eV for the oxygen 1s state. Generally, we found that the ev*GW*_0_ shifts get progressively larger with increasing BE. Therefore, we propose here a modification of the Hedin approach, where we determine an *n*-specific shift Δ*H*_*n*_ for the (core) state *n* of interest. Δ*H*_*n*_ is determined as
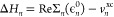
12and the QP equation transforms then to
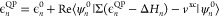
13Δ*H*_*n*_ is still a global shift, which is, however, specific for the respective (core) state of interest. For example, to obtain ϵ_C1s_^QP^ for the CO molecule, we solve eq 13 with Δ*H*_C1s_, whereas we solve it with Δ*H*_O1s_ for ϵ_O1s_^QP^. In the case of HCOOH, we determine Δ*H*_*n*_ for each O separately.

**Figure 1:**
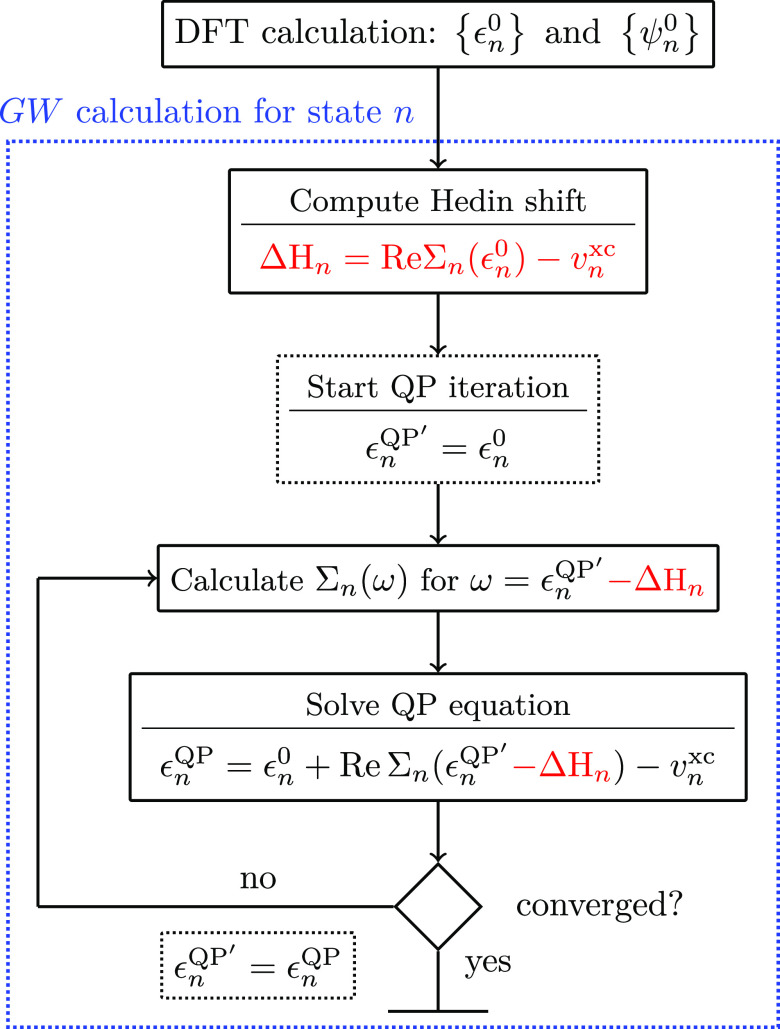
Flowchart for the *G*_Δ*H*_*W*_0_ scheme starting from a KS-DFT calculation. The additional terms with respect to a *G*_0_*W*_0_ calculation are highlighted in red.

The flowchart of a *G*_Δ*H*_*W*_0_ calculation is shown in Figure 1. In a *G*_Δ*H*_*W*_0_ calculation of state *n*, we calculate Δ*H*_*n*_ once, insert it in eq 13, and iterate the latter as in a regular *G*_0_*W*_0_ calculation. The shift Δ*H*_*n*_ is kept constant during the iteration of the QP equation. Compared to a *G*_0_*W*_0_ calculation, the computation of Δ*H* is the only computational overhead that we introduce. The computational cost of a *G*_Δ*H*_*W*_0_ calculation is thus practically the same as for a *G*_0_*W*_0_ calculation.

We point out that the Hedin shift is not an arbitrary scissor shift of the KS eigenvalues {ϵ_*n*_^0^}, where the energies of the occupied and virtual states are shifted down and up, respectively. (i) The eigenvalues {ϵ_*n*_^0^} are still used to construct *W*_0_. The shift is only applied in *G,* and its sign is, unlike in a scissor shift approach, independent on the occupation of state *m* in eq 9. (ii) More importantly, the expression for Δ*H*_*n*_ is derived by enforcing self-consistency for the energy of state *n*. The motivation for the Hedin shift and its derivation was comprehensively discussed for the valence case in the literature^74,77,79^ and is therein also referred to as “alignment of the chemical potential” or “adjusting the energy scale of *G*_0_”.

The Hedin shift can be understood as the simplest step toward self-consistency and viewed as one-diagonal element correction in the context of the ev*GW*_0_ method. In ev*GW*_0_, we replace the KS energies ϵ_*m*_^0^ in eq 3 by ϵ_*m*_^QP^ = ϵ_*m*_^0^ + Δϵ_*m*_, see eq 8, where *m* is an index that runs over all occupied and virtual states and Δϵ_*m*_ is the *GW* correction. In the *G*_Δ*H*_*W*_0_ approach that we propose here, we aim to approximate the element Δϵ_*m*_, which has the largest contribution, by Δ*H*, and neglect the rest. We found that the element Δϵ_*m*=*n*_, where *n* is the state of interest, is (by far) the most relevant one. This is the motivation for determining a state-specific shift Δ*H*_*n*_ and using eq 12 instead of eq 11 for our core-level calculations.

### 2.4 RSEs in RPA

The RS Green’s function approach is based on the same idea as the RSE corrections in RPA. Since we consider the introduction of the RS concept more illustrative for total than QP energies, we will briefly summarize the key equations derived by Ren et al.^68,69,80^ in the context of Rayleigh–Schrödinger perturbation theory (RSPT), before proceeding with the RS Green’s function approaches in Section 2.5.

In RSPT, the interacting *N*-electron Hamiltonian *Ĥ* is partitioned into a noninteracting mean-field Hamiltonian  and an interacting perturbation .
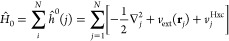
14
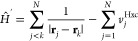
15where  is the single-particle Hamiltonian of the mean-field reference. The Hamiltonian  includes the kinetic term, the external potential *v*_ext_, and the mean-field potential *v*^Hxc^. The latter can be the single-particle potential from HF or KS-DFT and contains the Hartree and exchange–correlation terms. Following standard perturbation theory, single excitations (SEs) contribute to the second-order correlation energy and are given by^68,69^
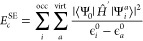
16
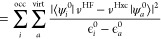
17where |Ψ_0_⟩ is the Slater determinant for the ground-state configuration and  for the singly excited configuration. The orbitals ψ_*i*/*a*_^0^ and corresponding orbital energies ϵ_*i*/*a*_^0^ are the ones of the  operator. The derivation of eq 17 from eq 16 is given in detail in the Supporting Information of ref (68). As evident from eq 17, the single correction vanishes if *v*^Hxc^ is the HF mean-field potential, which is a consequence of Brillouin’s theorem.^81^

**Figure 2:**
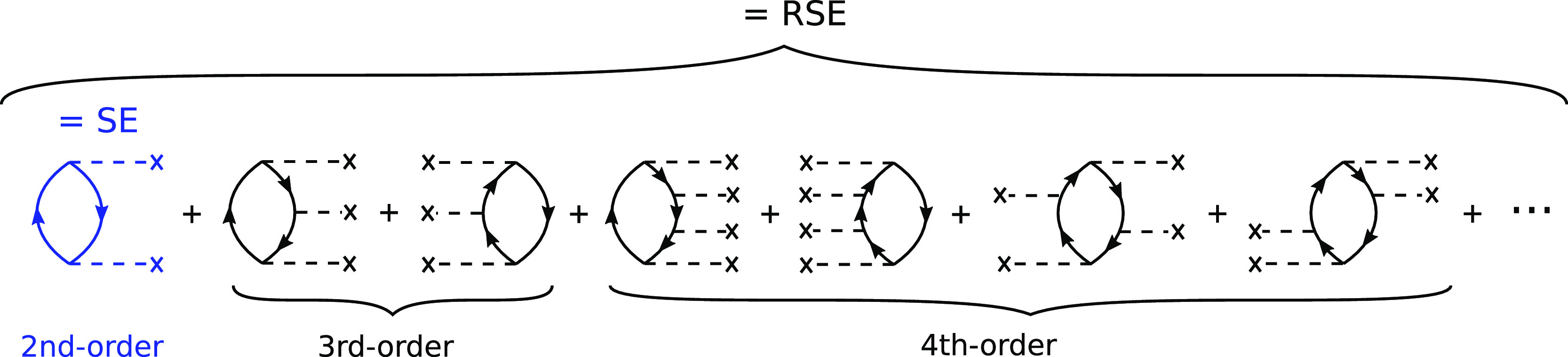
Goldstone diagrammatic for the RSE contributions. Dashed lines, which end with a cross, denote the matrix elements ⟨ψ_*p*_|*v*^HF^ – *v*^Hxc^|ψ_*q*_⟩.

The energy *E*_*c*_^SE^ is only the second-order correction to the correlation energy, as shown in Figure 2. The infinite summation of the higher-order diagrams yields the RSE correction. The derivation of the RSE correlation energy is given in detail in ref (69). To summarize briefly the procedure, the Fock matrix is evaluated with the KS orbitals. Subsequently, the occupied and unoccupied blocks of this matrix are diagonalized separately (subspace diagonalization), which yields a new set of (RS) eigenvalues and orbitals. Replacing ψ_*i*/*a*_ and ϵ_*i*/*a*_ in eq 17 with the RS eigenvalues and orbitals yields the RSE correlation energy.

### 2.5 RS Green′s Function *GW* Approaches

In analogy to the RPA RSE correction, the RS Green’s function *G*_RS_ is designed as an effective noninteracting reference system that includes all the single contributions. The RS Green’s function is defined as
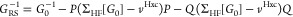
18where  is the projection into the occupied orbital space and *Q* = I – *P* is the projection into the virtual orbital space. Σ_HF_[*G*_0_] refers to a HF-like self-energy constructed with *G*_0_, which is usually the KS Green’s function. Σ_HF_ is the sum of the Hartree self-energy Σ^H^ and the exchange self-energy Σ^*x*^, that is, Σ_HF_ = Σ^H^ + Σ^*x*^, where  and . Note that both are built with the mean-field orbitals ψ_*n*_^0^ provided by, for example, KS-DFT. *v*^Hxc^ is the single-particle Hartree-exchange–correlation potential defined in eq 14. If the potential *v*^Hxc^ is the one from HF and if *G*_0_ is the HF Green’s function, then the second and third terms on the right-hand side of eq 18 vanish and *G*_RS_ corresponds to the HF Green’s function, which is again a consequence of Brillouin’s theorem. *G*_RS_ includes the single contributions, which are one source of the starting point dependence. As we showed previously,^67^ the dependence on the DFA is therefore reduced in the *G*_RS_*W*_0_ scheme. As for *G*_0_*W*_0_, the primary use case of the RS approach are closed-shell systems for the same reasons discussed in Section 2.1 and due to the restriction of Brillouin’s theorem to the closed-shell case. While not part of this work, we expect nevertheless that simple spin splittings as observed for 1s excitations can be captured by the RS approach.

The RS Green’s function is given as the solution of the two projected equations in the occupied orbital subspace^67^
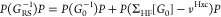
19and the virtual orbital subspace
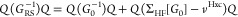
20

In practice, *G*_RS_ is obtained by a similar subspace diagonalization procedure as used for the RSE total energy corrections. The KS density matrix is used to construct the HF Hamiltonian , which defines the RS Hamiltonian . The equations for the occupied
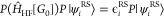
21and virtual subspace
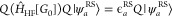
22are diagonalized separately.^67^ The subspace diagonalization yields the RS eigenvalues ϵ_*n*_^RS^ and corresponding eigenvectors *ψ*_*n*_^RS^ and is performed only once. The RS Hamiltonian can be reduced to the HF_diag_ method^38^ if only diagonal elements in eqs 21 and 22 are used. It has been shown that the *G*_0_*W*_0_(HF_diag_) method predicts accurate valence QP energies of molecular systems.^38^

The RS Green’s function is computed with the RS eigenvalues and orbitals
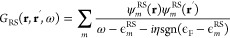
23and is diagonal in the occupied subspace and the virtual subspace.

In the *G*_RS_*W*_0_ approach,^67^ the RS Green’s function is used as a new starting point and the screened interaction is calculated with the KS Green’s function. The correlation part of the *G*_RS_*W*_0_ self-energy is^67^
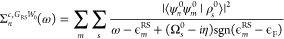
24where ρ_*s*_^0^ and Ω_*s*_^0^ are the transition densities and the RPA excitation energies calculated with the KS Green’s function. Then, the QP equation for *G*_RS_*W*_0_ is
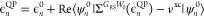
25Note that eqs 24 and 25 are slightly different from the original QP equation for *G*_RS_*W*_0_,^67^ where we used the RS eigenvectors ψ_*n*_^RS^. Here, we use for simplicity the KS instead of the RS orbitals because we found that the difference induced upon using RS orbitals is marginal.^67^ Since we use the KS orbitals, the exchange part of the *G*_RS_*W*_0_ self-energy is the same as in *G*_0_*W*_0_, see eq 5.

In this work, we introduce a new approach that uses the RS Green’s function as a new starting point and also calculates the screened Coulomb interaction with the RS Green’s function, denoted as *G*_RS_*W*_RS_. This means that *W*_RS_ is obtained by inserting the RS Green’s function into the RPA equation. Similar to *G*_RS_*W*_0_, the exchange part of the *G*_RS_*W*_RS_ self-energy is also the same as for *G*_0_*W*_0_, but the correlation part of the *G*_RS_*W*_RS_ self-energy becomes
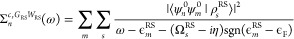
26where ρ_*s*_^RS^ and Ω_*s*_^RS^ are the transition densities and the RPA excitation energies calculated with the RS Green’s function. Then, the QP equation for the *G*_RS_*W*_RS_ approach follows as
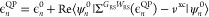
27The construction of the RS Green’s function scales only as  with respect to system size *N*. The overall scaling of *G*_RS_*W*_RS_ depends on the frequency integration technique that is used, which will be discussed more in detail in Section 4.4.

## 3 Computational Details

Core-level calculations were performed at the *G*_0_*W*_0_, ev*GW*_0_, ev*GW*, *G*_Δ*H*_*W*_0_, and *G*_RS_*W*_RS_ levels for the CORE65 benchmark set,^49^ which contains 65 1s excitation energies of 32 small molecules (30 × C 1s, 21 × O 1s, 11 × N 1s, and 3 × F 1s). Geometries and experimental reference values were taken from ref (49). Additionally, we studied the ETFA molecule. The structure of the latter was obtained upon request from the authors of ref (18) and is available in the Supporting Information.

The *G*_0_*W*_0_, ev*GW*_0_, ev*GW,* and *G*_Δ*H*_*W*_0_ calculations were carried out with the FHI-aims program package,^82,83^ which is based on numeric atom-centered orbitals (NAOs). The *G*_0_*W*_0_ and ev*GW*_0_ data were extracted from our previous work,^49^ while the ev*GW* and *G*_Δ*H*_*W*_0_ data were generated for this benchmark study. In the FHI-aims calculations, the contour deformation technique^22,31,84^ is used to evaluate the self-energy using a modified Gauss–Legendre grid^39^ with 200 grid points for the imaginary frequency integral part. The *G*_RS_*W*_RS_ calculations were performed with the QM4D program.^85^ In QM4D, the *GW* self-energy integral is calculated fully analytically, see eq 6. In FHI-aims and also in QM4D, the QP equation is always solved iteratively.

For ev*GW*_0_, ev*GW,* and *G*_Δ*H*_*W*_0_, we used the Perdew–Burke–Ernzerhof (PBE)^86^ functional for the underlying DFT calculation, while the *G*_0_*W*_0_ calculations employ the PBEh(α) hybrid functional^87^ with 45% exact exchange (α = 0.45). The *G*_RS_*W*_RS_ calculations were performed with PBE and three different hybrid functionals, namely, PBE0,^88,89^ PBEh(α = 0.45), and B3LYP.^90,91^ Note that PBE0 corresponds to PBEh(α = 0.25).

All *GW* results are extrapolated to the complete basis set limit using the Dunning basis set family cc-pV*n*Z.^92,93^ For a discussion of the basis set choice, we refer the reader to ref (52) and theSupporting Information of ref (50). Following ref (49), the extrapolation is performed by a linear regression with respect to the inverse of the total number of basis functions. A four-point extrapolation with *n* = 3–6 is performed for *G*_0_*W*_0_, ev*GW*_0_, ev*GW*, and *G*_Δ*H*_*W*_0_. For *G*_RS_*W*_RS_, we use only two points (*n* = 3, 4) due to computational limitations. We verified that this two-point extrapolation deviates only by 0.1 eV from the four-point scheme on average. The cc-pV*n*Z family consists of contracted Gaussian-type orbitals (GTOs), which can be considered as a special case of an NAO and are treated numerically in FHI-aims. Note that the cc-pV*n*Z basis sets are treated as spherical GTOs in FHI-aims, whereas in QM4D, they are processed as pure Cartesian GTOs. Both codes use the resolution-of-the-identity (RI) approach with the Coulomb metric (RI-V).^94^ In FHI-aims, the RI auxiliary basis sets are generated on-the-fly as described in ref (39). For the QM4D calculations, the corresponding RI basis sets for cc-pVTZ and cc-pVQZ from ref (95) were used.

Relativistic effects were included for all calculations as post-processing step following the approach in refs (49) and (50); that is, we performed a nonrelativistic *GW* calculation on top of a nonrelativistic KS-DFT calculation and added the corrective term derived in ref (50) to the *GW* QP energies. The magnitude of the corrections increases with the atomic number and ranges from 0.12 eV for C 1s to 0.71 eV for F 1s. The relativistic corrections were derived for a free neutral atom at the PBE level and were obtained by evaluating the difference between the 1s eigenvalues from the radial KS and the 4-component Dirac-KS equation.

## 4 Results and Discussion

### 4.1 Solution Behavior

**Figure 3:**
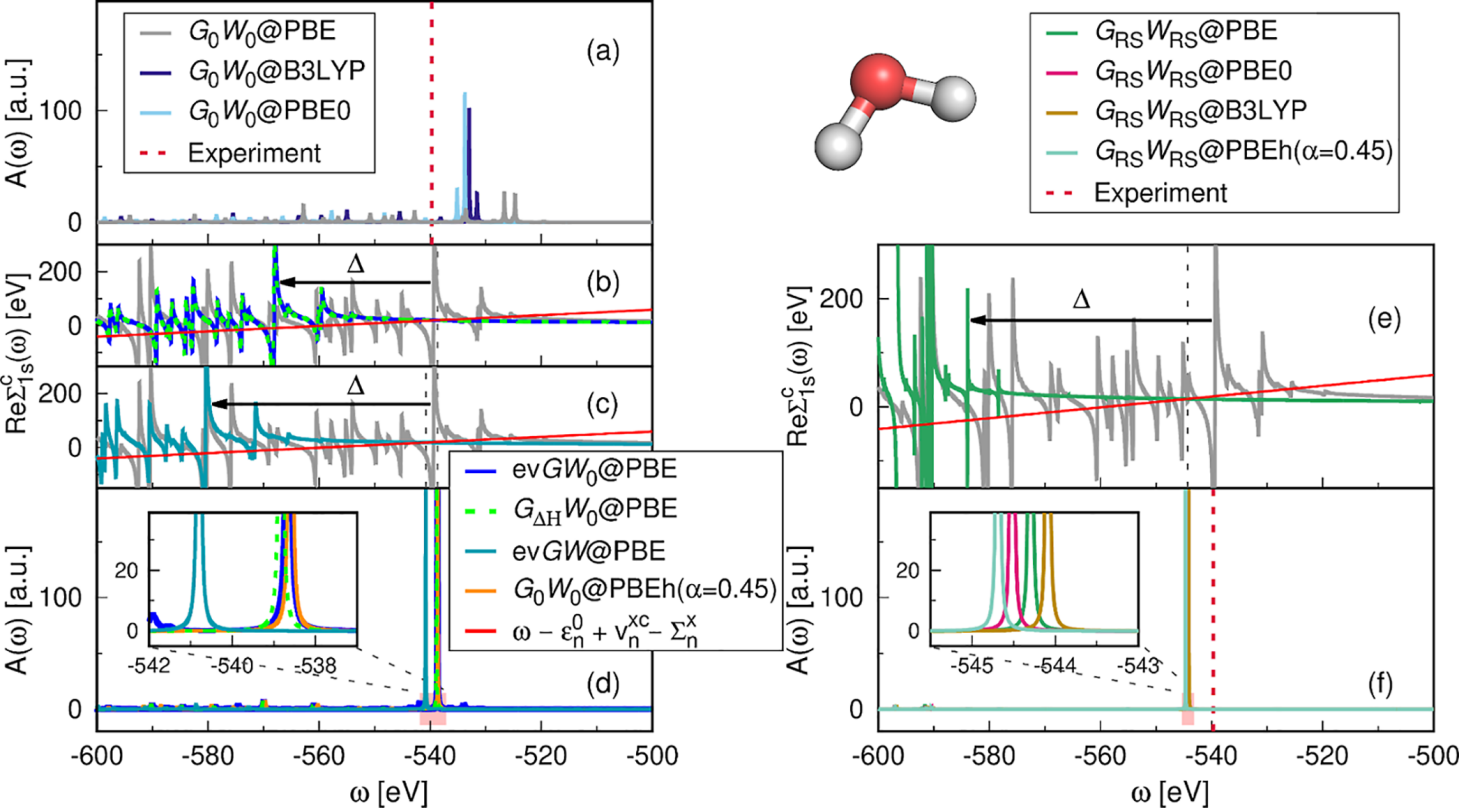
O 1s excitation for a single water molecule from *G*_0_*W*_0_, ev*GW*_0_, *G*_Δ*H*_*W*_0_, ev*GW*, and *G*_RS_*W*_RS_ computed at the cc-pVQZ level. (a) Spectral function *A*(ω) (eq 7) from *G*_0_*W*_0_ using starting points with no exact exchange or a low amount. (b,c) Real part of the correlation self-energy Σ^*c*^(ω) in ev*GW*_0_*@*PBE, *G*_Δ*H*_*W*_0_, and ev*GW@*PBE. Diagonal matrix elements  for the oxygen 1s orbital. The intersection with the red line is the graphical solution of eq 1. The vertical gray-dashed line indicates the QP solution of this graphical solution, and Δ indicates the shift with respect to *G*_0_*W*_0_*@*PBE (gray). (d) Spectral functions in ev*GW*_0_*@*PBE, *G*_Δ*H*_*W*_0_*@*PBE, ev*GW@*PBE, and *G*_0_*W*_0_*@*PBEh(α = 0.45). (e) Self-energy matrix elements ReΣ_*n*_^*c*^(ω) in *G*_RS_*W*_RS_*@*PBE (green) compared to *G*_0_*W*_0_*@*PBE (gray). (f) Spectral functions *A*(ω) in *G*_RS_*W*_RS_ with different starting points.

In our previous work,^49^ we showed that standard *G*_0_*W*_0_ calculations starting from a GGA functional, which are routinely applied to valence states, lead to an erroneous multi-solution behavior for deep core states. It is thus important to confirm that the respective *GW* flavors yield indeed a unique solution. Only looking at the QP energies obtained by iterating eq 1 is typically not enough to verify the latter. Detailed insight into the solution behavior of *GW*-based methods can be obtained by (i) plotting the real part of the correlation self-energy Σ^*c*^ and (ii) plotting the spectral function *A*(ω) as defined in eq 7. In Figure 3, we investigate *A*(ω) and the diagonal matrix elements Σ_*n*_^*c*^(ω) for the 1s oxygen orbital of a single water molecule. Results are shown for *G*_0_*W*_0_ and *G*_RS_*W*_RS_ with different starting points [PBE, PBE0, B3LYP, PBEh(α = 0.45)] as well as partial self-consistent schemes, namely, ev*GW*_0_, ev*GW*, and *G*_Δ*H*_*W*_0_, using PBE for the underlying DFT calculation.

We start our discussion with the *G*_0_*W*_0_ spectral functions and self-energy elements displayed in Figure 3a,b,d, where we reproduced for convenience the *G*_0_*W*_0_*@*PBE, *G*_0_*W*_0_*@*PBE0, and *G*_0_*W*_0_*@*PBEh(α = 0.45) results, which are also presented in ref (49). Figure 3b shows the self-energy from a *G*_0_*W*_0_*@*PBE calculation (gray line), which exhibits many poles. The poles are broadened by the η-term in eq 3 and thus appear as spikes in the self-energy. For *G*_0_*W*_0_*@*PBE, we find that the poles are located in the frequency region where the QP solution is expected (around −540 eV). As already outlined in Section 2.1, we can obtain the graphical solution of eq 1 by finding the intersections with the straight line ω – ε_*n*_^0^ + *v*_*n*_^*xc*^ – Σ_*n*_^*x*^. For *G*_0_*W*_0_, we observe many intersections, which are all valid solutions of the QP equation. The corresponding spectral function in Figure 3a shows many peaks with equal spectral weight but no clear main peak that could be assigned to the QP excitation in contrast to the experiment, where a sharp peak at 539.7 eV^4^ is observed. A main peak starts to emerge for hybrid functional starting points, such as PBE0 (α = 0.25) and B3LYP (α = 0.20). However, *G*_0_*W*_0_*@*PBE0 and *G*_0_*W*_0_*@*B3LYP still yield an unphysical second peak, which carries a large fraction of the spectral weight.

As already discussed in ref (49), the reason for this unphysical behavior is the overlap of the satellite spectrum with the QP peak. Satellites are due to multielectron excitations that accompany the photoemission process, for example, shake-up satellites, which are produced when the core photoelectron scatters a valence shell electron to a higher unoccupied energy level.^96,97^ These peaks appear as series of smaller peaks at higher BEs than the QP energy. For molecules, the spectral weight of these peaks is orders of magnitudes smaller than for the main excitation.^97^ Satellites occur in frequency regions where the real part of the self-energy has poles. As demonstrated in, for example, ref (31), the imaginary part of the self-energy exhibits complementary peaks at these frequencies (Kramers–Kronig relation). According to eq 7, large imaginary parts lead to peaks with small spectral weight, that is, peaks with a satellite character.

The occurrence of pole features around the QP excitations for deep core states can best be understood by analyzing the denominator of the fully analytic expression of the self-energy given in eq 6. Σ_*n*_^*c*^(ω) has poles on the real axis for η → 0 at ϵ_*i*_^0^ – Ω_*s*_ (occupied states *i*) and ϵ_*a*_^0^ + Ω_*s*_ (virtual states *a*). For occupied states, the eigenvalues ϵ_*i*_^0^ are too large (too positive), and the charge neutral excitations Ω_*s*_ are underestimated at the PBE level. As a result, the poles ϵ_*i*_^0^ – Ω_*s*_ are at too positive frequencies and the satellite thus too close to the QP peak. For virtual states, the same reasoning holds for the poles ϵ_*a*_^0^ + Ω_*s*_ but with reversed sign, that is, the poles are at too small frequencies. The separation between satellites and QP peak is also too small for valence excitations. However, the problem gets progressively worse further away from the Fermi level since the absolute differences between PBE eigenvalues ϵ_*i*_^0^ and the QP excitation increase. We demonstrated this for semicore states,^58^ for which a distinct QP peak is still obtained. However, for deep core states, the separation becomes finally so small that the satellites merge with the QP peak.

The proper separation between the QP excitation and satellites can be restored by using an ev*GW*_0_ scheme. The ev*GW*_0_*@*PBE self-energy is shown in Figure 3b (reproduced from ref (49)). Iterating the eigenvalues in *G* shifts the on-set of the pole structure too more negative frequencies. The overall pole structure is very similar to *G*_0_*W*_0_*@*PBE but shifted by a constant value of Δ = −28.7 eV. The *G*_Δ*H*_*W*_0_*@*PBE self-energy displayed in Figure 3b is almost identical to ev*GW*_0_*@*PBE. The shift of the pole structure compared to *G*_0_*W*_0_*@*PBE is with Δ = −28.8 eV only slightly larger than for ev*GW*_0_. The rigid Δ shift of the pole features can be understood as follows: in ev*GW*_0_ and *G*_Δ*H*_*W*_0_, the KS eigenvalue ϵ_*m*_^0^ is replaced with ϵ_*m*_^0^ + Δϵ_*m*_ and ϵ_*m*_^0^ + Δ*H*_1s_, respectively, where Δϵ_*m*_ is the self-consistent *GW* correction for state *m* and Δ*H*_1s_ its non-self-consistent approximation for the O 1s state (see eq 12). The poles are consequently located at ϵ_1s_^0^ + Δϵ_1s_ – Ω_*s*_ and ϵ_1s_^0^ + Δ*H*_1s_ – Ω_*s*_. Since both corrections, Δϵ_1s_ and Δ*H*_1s_, are negative for PBE starting points, the poles, that is, satellites, move to more negative frequencies and are properly separated from the main excitation. The spectral function now exhibits a distinct QP peak as evidenced by Figure 3d.

While the main effect of ev*GW*_0_ and *G*_Δ*H*_*W*_0_*@*PBE for deep core levels is the correction of the spurious transfer of spectral weight to the satellites, it affects also the location of the QP peak. This has been graphically demonstrated for molecular valence states in Figure 25 of ref (31): by shifting the pole structure to more negative frequencies, the slope of ReΣ_*n*_^*c*^ will be flatter in the region where the QP excitation is expected. As a consequence, the intersection with the straight line ω – ϵ_*n*_^0^ + *v*_*n*_^*xc*^ – Σ_*n*_^*x*^ (= QP solution) will be at more negative frequencies compared to *G*_0_*W*_0_*@*PBE.

As discussed in detail previously,^49^ the effect of eigenvalue self-consistency can be mimicked in a *G*_0_*W*_0_ calculation by using a hybrid functional with a high amount of exact exchange α. We showed that increasing α progressively shifts the pole features to more negative frequencies. For α = 0.45, the ev*GW*_0_*@*PBE self-energy is approximately reproduced, and the spectral function shows a distinct peak as displayed in Figure 3d. We note here again that values of α < 0.3 do not yield a clear main peak^49^ and thus no unique solution, which is also demonstrated in Figure 3a.

The ev*GW* approach and the *G*_RS_*W*_RS_ schemes lead to a significantly stronger shift of the pole features than ev*GW*_0_*@*PBE or *G*_Δ*H*_*W*_0_*@*PBE, as shown in Figure 3c,e. The spectral functions displayed in Figure 3d,f confirm that ev*GW* and *G*_RS_*W*_RS_ yield a distinct peak in the spectrum. The RS eigenvalues of the occupied orbitals are more negative, and the ones of the virtual orbitals are more positive than the KS eigenvalues. In addition, RPA evaluated with RS fundamental gaps provides larger excitation energies Ω_*s*_. In *G*_RS_*W*_RS_, the poles at ϵ_*a*_ + Ω_*s*_ are shifted in the positive direction and the poles at ϵ_*i*_ – Ω_*s*_ are shifted in the negative direction. Therefore, satellites from these poles are separated from the main peak. For *G*_RS_*W*_RS_, a unique solution is obtained for all starting points. As shown in Figure S1 (see Supporting Information), the *G*_RS_*W*_0_ approach suffers from a multi-solution behavior in the deep core region and cannot be applied for core-level calculations.

### 4.2 CORE65 Benchmark

**Figure 4:**
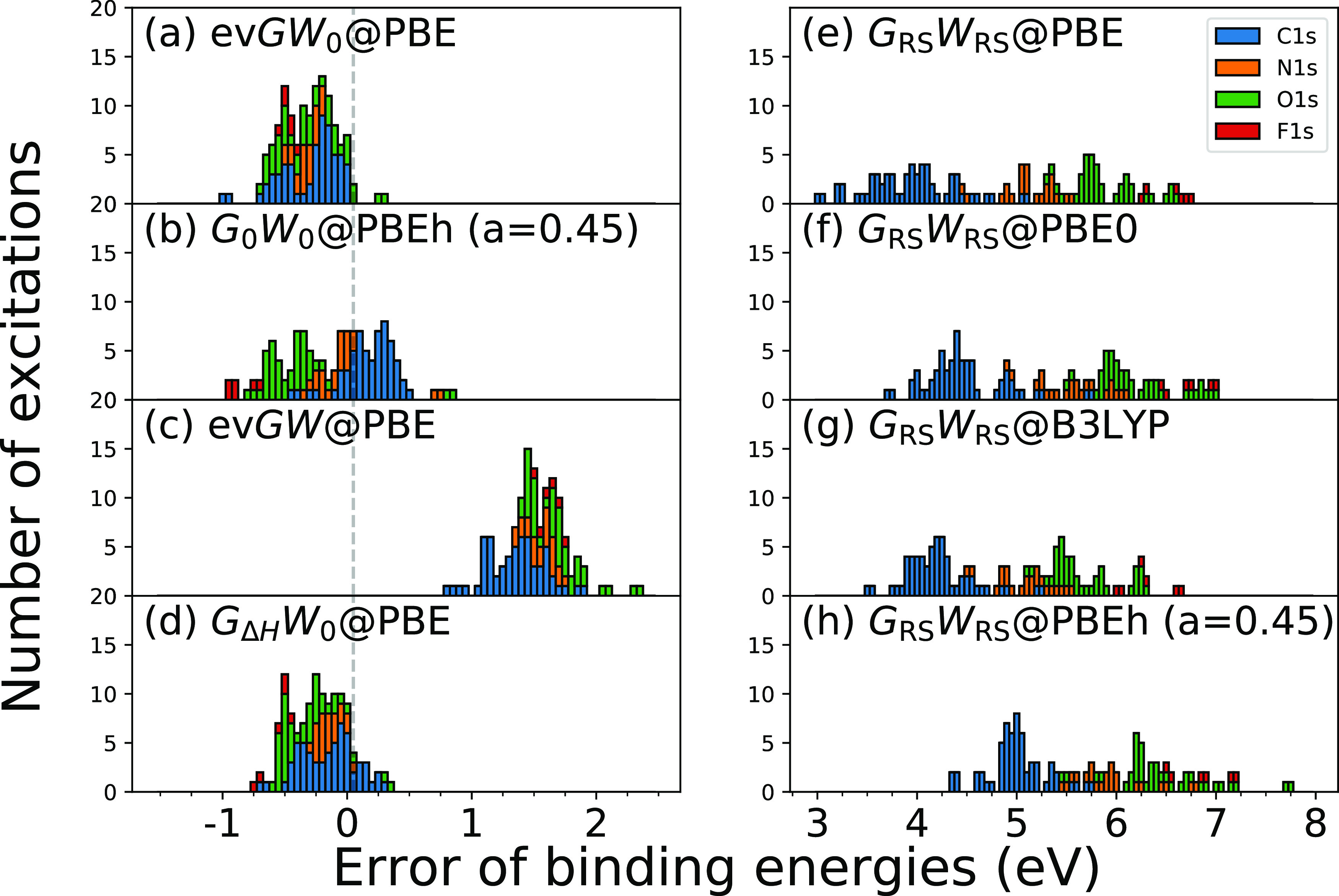
Distribution of errors with respect to the experiment for absolute 1s BEs of the CORE65 benchmark set, where the error is defined as error_*i*_ = CLBE_*i*_^theory^ – CLBE_*i*_^exp^. The histogram is stacked. Eight *GW* approaches are compared: (a) ev*GW*_0_@PBE, (b) *G*_0_*W*_0_@PBEh(α = 0.45), (c) ev*GW*@PBE, (d) *G*_Δ*H*_*W*_0_@PBE, (e) *G*_RS_*W*_RS_@PBE, (f) *G*_RS_*W*_RS_@PBE0, (g) *G*_RS_*W*_RS_@B3LYP, and (h) *G*_RS_*W*_RS_@PBEh(α = 0.45).

**Figure 5:**
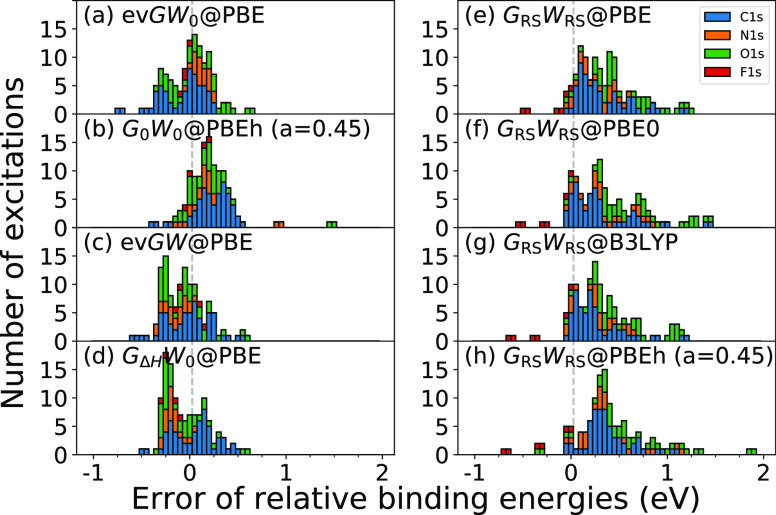
Distribution of errors with respect to the experiment for relative 1s BEs of the CORE65 benchmark set. The histogram is stacked. CH_4_, NH_3_, H_2_O, and CH_3_F have been used as reference molecules for C 1s, N 1s, O 1s, and F 1s, respectively. Eight *GW* approaches are compared: (a) ev*GW*_0_@PBE, (b) *G*_0_*W*_0_@PBEh(α = 0.45), (c) ev*GW*@PBE, (d) *G*_Δ*H*_*W*_0_@PBE, (e) *G*_RS_*W*_RS_@PBE, (f) *G*_RS_*W*_RS_@PBE0, (g) *G*_RS_*W*_RS_@B3LYP, and (h) *G*_RS_*W*_RS_@PBEh(α = 0.45).

In the following, we discuss the CORE65 results for the *GW* schemes for which a physical solution behavior was confirmed in Section 4.1, namely, ev*GW*_0_*@*PBE, ev*GW@*PBE, *G*_0_*W*_0_*@*PBEh(α = 0.45), *G*_Δ*H*_*W*_0_*@*PBE, and *G*_RS_*W*_RS_ with four different starting points [PBE, PBE0, B3LYP, and PBEh(α = 0.45)]. The distribution of the errors with respect to experiment is shown in Figures 4 and 5 for the absolute CLBEs and the relative CLBEs, respectively. The corresponding MAE and the mean errors (MEs) are given in Table 1. The error of excitation *i* is defined as error_*i*_ = CLBE_*i*_^theory^ – CLBE_*i*_^experiment^.

**Table 1:** MAE and ME in eV with Respect to Experiment for Absolute and Relative CLBEs of the CORE65 Benchmark Set^a^

	ev*GW*_0_*@*		*G*_0_*W*_0_*@*		ev*GW@*		*G*_Δ*H*_*W*_0_*@*		*G*_RS_*W*_RS_*@*							
	PBE		PBEh		PBE		PBE		PBE		PBE0		B3LYP		PBEh	
core level	MAE	ME	MAE	ME	MAE	ME	MAE	ME	MAE	ME	MAE	ME	MAE	ME	MAE	ME
absolute CLBEs																
all	0.30	–0.29	0.33	–0.08	1.53	1.53	0.25	–0.20	4.88	4.88	5.32	5.32	4.94	4.94	5.72	5.72
C 1s	0.27	–0.27	0.24	0.19	1.37	1.37	0.20	–0.13	3.97	3.97	4.50	4.50	4.24	4.24	5.03	5.03
N 1s	0.30	–0.30	0.16	–0.01	1.58	1.58	0.14	–0.13	5.13	5.13	5.59	5.59	5.12	5.12	6.00	6.00
O 1s	0.32	–0.28	0.48	–0.40	1.70	1.70	0.35	–0.31	5.91	5.91	6.24	6.24	5.71	5.71	7.48	7.48
F 1s	0.44	–0.44	0.83	–0.83	1.65	1.65	0.54	–0.54	6.56	6.56	6.75	6.75	6.32	6.32	6.88	6.88
Relative CLBEs																
all	0.18	0.02	0.26	0.23	0.18	–0.03	0.19	–0.02	0.40	0.39	0.43	0.43	0.37	0.36	0.48	0.46
C 1s	0.18	–0.05	0.29	0.25	0.19	–0.01	0.20	0.07	0.36	0.36	0.33	0.33	0.30	0.30	0.41	0.41
N 1s	0.14	0.14	0.23	0.21	0.14	–0.13	0.16	–0.15	0.29	0.29	0.39	0.39	0.30	0.30	0.40	0.40
O 1s	0.22	0.08	0.25	0.24	0.18	–0.03	0.20	–0.06	0.56	0.56	0.66	0.66	0.56	0.56	0.68	0.65
F 1s	0.05	–0.05	0.11	0.10	0.11	0.02	0.16	–0.16	0.16	–0.10	0.16	0.00	0.20	0.02	0.22	0.00

a The error for excitation *i* is defined as error_*i*_ = CLBE_*i*_^theory^ – CLBE_*i*_^exp^. The relative CLBEs are the shifts with respect to a reference molecule, ΔCLBE = CLBE – CLBE_ref_mol_. CH_4_, NH_3_, H_2_O, and CH_3_F have been used as reference molecules for C 1s, N 1s, O 1s, and F 1s, respectively.

Starting the discussion with the absolute CLBEs, we find that ev*GW*_0_@PBE, *G*_0_*W*_0_@PBEh(α = 0.45), and *G*_Δ*H*_*W*_0_@PBE yield the best results with error distributions close to zero and MAEs of ≈0.3 eV. The smallest overall MAE of 0.25 eV is obtained with *G*_Δ*H*_*W*_0_@PBE. Figure 4a,d shows that the errors from ev*GW*_0_*@*PBE and *G*_Δ*H*_*W*_0_@PBE are tightly distributed but mostly negative; that is, the computed CLBEs are slightly underestimated. Generally, we find that the *G*_Δ*H*_*W*_0_@PBE scheme reproduces the ev*GW*_0_*@*PBE results almost perfectly. The slight underestimation of the absolute CLBEs by ev*GW*_0_*@*PBE and *G*_Δ*H*_*W*_0_@PBE might be due to insufficiencies in the cc-pV*n*Z basis sets, which are not captured by the extrapolation procedure. A very recent study^52^ with *G*_0_*W*_0_*@*PBEh showed that increasing the number of core functions by, for example, uncontracting the cc-pV*n*Z basis sets yields larger absolute CLBEs. The reported increase is in the range of 0.25 to maximal 0.5 eV, indicating that the CLBEs from ev*GW*_0_ and *G*_Δ*H*_*W*_0_@PBE might be even closer to experiment with core-rich basis sets.

The error distribution of *G*_0_*W*_0_@PBEh(α = 0.45), which is displayed in Figure 4b, is centered around zero, yielding also the smallest overall ME, see Table 1. Compared to ev*GW*_0_*@*PBE and *G*_Δ*H*_*W*_0_@PBE, the spread of the *G*_0_*W*_0_*@*PBEh errors is larger and a clustering by species can be observed. The C 1s BEs are overestimated, while N 1s, O 1s, and F 1s are increasingly underestimated. This is due to the species dependence of the α parameter, which we discussed in refs (49) and (50). As we showed in ref (50), including relativistic effects reduces the species dependence of α but does not remove it completely. The optimal α value, α_opt_, increases from 0.44 (C 1s) to 0.49 (F 1s), after including relativistic corrections. For α < α_opt_, the CLBEs are too small and for α > α_opt_ too large. As a result, the C 1s BEs are overestimated for α = 0.45, and O 1s and F 1s BEs are underestimated.

ev*GW*@PBE systematically overestimates the absolute CLBEs by 1–2 eV since iterating also the eigenvalues in *W* effectively leads to an underscreening. This underscreening is also expected for the higher-level self-consistency schemes, such as sc*GW*^63^ and qs*GW*,^64^ and is due to the missing vertex correction. The performance of ev*GW@*PBE for deep core levels seems to be comparable to qs*GW*. An exploratory study by van Setten et al.^47^ reported that qs*GW* overestimates the absolute 1s BEs of small molecules by 2 eV. This indicates that qs*GW* suffers from similar underscreening effects and that the orbitals inserted in the *GW* scheme have a minor effect on the core-level QP energies. The success of ev*GW*_0_*@*PBE on the contrary is based on a fortuitous error cancellation effect, which can be explained as follows: at the PBE level, the fundamental gap is underestimated and inserting the PBE eigenvalues in *W* consequently yields an overscreened potential. However, the overscreening in *W* compensates the underscreening introduced by the missing vertex corrections. Our observation for deep core levels agrees with previous work on valence states. Underscreening effects have been previously discussed for sc*GW* and qs*GW* for the *GW*100 benchmark set.^65^ Comparing ev*GW*_0_*@*PBE and ev*GW@*PBE for valence excitations, it was found that ev*GW*_0_ improves upon *G*_0_*W*_0_,^98,99^ while ev*GW* yields too large band gaps^98^ and overly stretched spectra.^100^ We acknowledge that the performance difference between ev*GW*_0_*@*PBE and ev*GW@*PBE is less pronounced for valence states (mostly <0.5 eV) and might be partly superimposed by a system dependence and numerical effects, for example, basis set convergence, which are in a similar range. For CLBEs on the contrary, we operate on energy scales which are an order of magnitude larger leading also to errors/performance differences which are an order of magnitude larger.

The error distributions for the absolute CLBEs from *G*_RS_*W*_RS_ are shown in Figure 4e–h for four different starting points. *G*_RS_*W*_RS_ overestimates the absolute CLBEs by 3–8 eV with an MAE between 5 and 6 eV. The reason for the large overestimation is that the RS fundamental gap is too large, which then leads, similarly as in *G*_0_*W*_0_*@*HF,^100^ to an underscreening in *W*. One way to reduce the underscreening is to include corrections for the electron correlation in the RS Hamiltonian, which is dominated by exchange interactions. An alternative strategy is to compensate the underscreening by including vertex corrections, for example, to use the *T*-matrix formalism,^30,71,72^ where the two-point screened interaction *W* is replaced with a four-point effective interaction *T*. However, methods such as the *T*-matrix are computationally much more expensive than *GW* due to their higher complexity. We recently applied the *G*_RS_*T*_RS_ scheme to the CORE65 benchmark set.^71^ Comparing *G*_RS_*W*_RS_ and *G*_RS_*T*_RS_, the overestimation is indeed reduced by *G*_RS_*T*_RS_, which yields an ME of ≈1.5 eV. However, the errors for the absolute CLBEs are still an order of magnitude larger than for computationally cheaper schemes such as ev*GW*_0_ or *G*_Δ*H*_*W*_0_, which rely on a very fortunate error cancellation effect that leads to a balanced screening.

Furthermore, we find that the overestimation with *G*_RS_*W*_RS_ increases with the atomic number, that is, from the C 1s to the F 1s excitations. This species dependence is inherited from the KS-DFT calculation. As shown in Table S8 in the Supporting Information, the deviations of the CLBEs obtained from KS-DFT eigenvalues  generally increase from C 1s to F 1s for all DFAs. We expect that, for example, adding correlation contributions to the RS Hamiltonian would also reduce this undesired species dependence.

The motivation of the RS approach is the reduction of the starting point dependence. As evident from Figure 4e–h, the large overestimation is observed for all DFAs. Based on the MAEs and MEs in Table 1, it can be shown that the starting point dependence is on average <1 eV for α = 0.0–0.45. As shown in Figure S2 (Supporting Information) for a single water molecule, ev*GW*_0_ seems to reduce the starting point dependence less. For the same α range, the CLBE changes by ≈2 eV. The direct comparison with *G*_0_*W*_0_ is difficult because a unique solution is only obtained for α > 0.3. However, we can study the change of the CLBEs for α = 0.3–1.0, see Figure S2 (Supporting Information), which shows that the starting point dependence is 10 eV with *G*_0_*W*_0_ compared to 2.8 eV with ev*GW*_0_.

Moving now to the relative CLBEs, we observe that ev*GW*_0_*@*PBE, *G*_0_*W*_0_*@*PBEh, ev*GW@*PBE, and *G*_Δ*H*_*W*_0_*@*PBE yield MAEs of ≈0.2–0.3 eV, and *G*_RS_*W*_RS_ MAEs of 0.4–0.5 eV, see Table 1. The errors of the relative CLBEs are centered and tightly distributed around zero for ev*GW*_0_*@*PBE, ev*GW@*PBE, and *G*_Δ*H*_*W*_0_*@*PBE. The perturbative schemes *G*_0_*W*_0_*@*PBEh and *G*_RS_*W*_RS_ slightly overestimate the relative CLBEs. The latter is evident from the positive MEs and the error distributions in Figure 4b,e–h, which are not centered at zero but exhibit a small offset toward positive values. The RS results show a larger spread compared to *G*_0_*W*_0_*@*PBEh and the partial self-consistent schemes. Furthermore, outliers with errors >1 eV are observed for *G*_0_*W*_0_*@*PBEh and in particular for *G*_RS_*W*_RS_. The largest outliers are primarily O 1s excitations, which originate from the underlying DFT calculation, as evident from Table S9 (see Supporting Information), which shows the MAEs of the relative CLBEs from the KS-DFT eigenvalues. For all four functionals (PBE, PBE0, B3LYP, and PBEh), we obtained the largest MAE at the KS-DFT level for the O 1s excitations. These errors are inherited in the one-shot *G*_0_*W*_0_ and *G*_RS_*W*_RS_ approaches because of their perturbative nature.

The chemical shifts between CLBEs of the same atomic type can be smaller than 0.5 eV for second row elements^4^ and even as small as 0.1 eV for C 1s.^101^ Therefore, the errors for absolute CLBEs from ev*GW* and *G*_RS_*W*_RS_ are too large to align or resolve experimental XPS spectra, for which reference data are not available. The most promising methods are ev*GW*_0_*@*PBE, *G*_0_*W*_0_*@*PBEh, and *G*_Δ*H*_*W*_0_*@*PBE. With MAEs between 0.2 and 0.3 eV for absolute and relative CLBEs, the accuracy is well within the chemical resolution required to interpret most XPS data. The disadvantage of the *G*_0_*W*_0_*@*PBEh(α) scheme is the need for tuning the α parameter. In addition, the species dependence of α_opt_ for C–F cannot be completely removed and is expected to increase for heavier elements. For example, we found α_opt_ = 0.61 for sulfur 1s excitations.^54^ Conversely, the accuracy of ev*GW*_0_*@*PBE and *G*_Δ*H*_*W*_0_*@*PBE is species independent. In addition, the already very good agreement of ev*GW*_0_@PBE and *G*_Δ*H*_*W*_0_*@*PBE with experiment might further improve with core-rich basis sets, as already mentioned before.

### 4.3 ETFA Molecule

**Figure 6:**
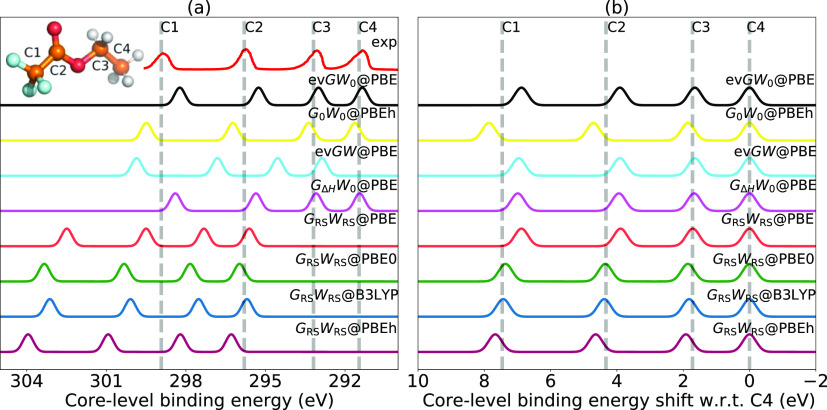
Comparison of the C 1s XPS spectrum of ETFA (a) for absolute CLBEs and (b) relative CLBEs obtained from ev*GW*_0_@PBE, *G*_0_*W*_0_@PBEh(α = 0.45), ev*GW*@PBE, *G*_Δ*H*_*W*_0_@PBE, *G*_RS_*W*_RS_@PBE, *G*_RS_*W*_RS_@PBE0, *G*_RS_*W*_RS_@B3LYP, and *G*_RS_*W*_RS_@PBEh(α = 0.45). Dashed lines indicate the experimental ref (102).

We further examine the performance of the eight *GW* approaches, which we applied to the CORE65 benchmark set in Section 4.2, for C 1s excitations of the ETFA molecule, which is also referred to as the “ESCA molecule” in the literature.^102^ The ETFA molecule was synthesized to demonstrate the potential of XPS for chemical analysis in the late 1960s. It contains four carbon atoms in various chemical environments, see the inset of Figure 6a. The ETFA molecule presents a challenge because of the extreme variations of the chemical shifts, which range up to 8.0 eV and decrease from the CF_3_ to the CH_3_ end. The four C 1s signals are separated by several electronvolts due to the widely different electronegativities of the substituents on the carbon atoms. ETFA is thus an important reference system and was very recently used to benchmark the performance of different functionals in ΔSCF calculations^18,104^ and *GW* approaches.^45^

In equilibrium, ETFA exists in two dominating conformations (anti-anti and anti-gauche); that is, each peak in the spectrum is a superposition of the signals from both conformers.^102^ However, including the different conformations is primarily important when resolving the vibrational profiles of the peaks, which is not the scope of our benchmarking effort. The experimental conformational shifts are <0.1 eV.^102^ To ensure direct comparability with the computational data from ref (18), we include thus only the anti-anti conformer.

The first high-quality experimental spectrum of the free ETFA molecule in gas phase was reported in 1973,^103^ while new results were published by Travnikova et al. in 2012,^102^ see Table 2. Both results were referenced in the most recent studies.^18,45^ The newer results have a higher resolution and are vibrationally resolved. More relevant for this work is that the chemical shifts of the more “descreened” carbon atoms are significantly smaller than in the older spectrum. The difference between the experimental spectra was attributed to missing correction techniques in early multi-channel plate detectors. We follow here the reasoning of ref (45), pointing out that coupled-cluster results^8^ are significantly closer to the new experimental data (in particular the chemical shifts). We use thus the data by Travnikova et al. as the experimental reference.

**Table 2:** Absolute C 1s CLBEs for the ETFA Molecule^a^

	C1	C2	C3	C4	ME	MAE
experiment^103^	299.45	296.01	293.07	291.20		
experiment^102^	**298.93**	**295.80**	**293.19**	**291.47**		
ev*GW*_0_*@*PBE	–0.70	–0.54	–0.19	–0.12	–0.36	0.36
*G*_0_*W*_0_*@*PBEh	0.56	0.54	0.30	0.16	0.39	0.39
*G*_Δ*H*_*W*_0_*@*PBE	–0.53	–0.44	–0.10	–0.02	–0.27	0.27
ev*GW@*PBE	0.93	1.01	1.35	1.40	1.17	1.17
*G*_RS_*W*_RS_*@*PBE	3.56	3.70	4.13	4.14	3.88	3.88
*G*_RS_*W*_RS_*@*PBE0	4.41	4.53	4.65	4.51	4.52	4.52
*G*_RS_*W*_RS_*@*B3LYP	4.20	4.29	4.33	4.23	4.26	4.26
*G*_RS_*W*_RS_*@*PBEh	5.02	5.13	5.02	4.82	5.00	5.00
ev*GW*_0_*@*PBE^45^	–0.41	–0.18	–0.04	–0.09	–0.18	0.18
ΔSCAN^18^	–0.15	–0.08	0.05	0.17	0.00	0.11
ΔCCSD(T)^8^	–0.35	–0.24	–0.31	–0.23	–0.28	0.28

a The deviation Δ_exp_ with respect to experiment and the corresponding ME and MAE are computed with the experimental data by Travnikova et al.^102^ (in bold), where error_*i*_ = CLBE_*i*_^theory^ – CLBE_*i*_^exp^. The labels of the C atoms are given in the inset of Figure 6a. For the @PBEh calculations, we use α = 0.45.

The comparison between the experimental spectrum and calculated spectra is shown in Figure 6. The differences to the experimental peak positions are reported in Tables 2 and 3. For the absolute CLBEs, the ETFA predictions are in line with the CORE65 benchmark results. ev*GW*_0_*@*PBE and *G*_Δ*H*_*W*_0_*@*PBE slightly underestimate the CLBEs, while ev*GW@*PBE and the RS schemes severely overestimate them, see Figure 6. *G*_0_*W*_0_*@*PBEh(α = 0.45) also overestimates the C 1s energies slightly. As discussed in Section 4.2, this is due to the fact that α_opt_ is slightly smaller than 0.45 for C 1s excitation. C 1s excitations are consequently slightly overestimated in *G*_0_*W*_0_*@*PBEh. *G*_Δ*H*_*W*_0_*@*PBE provides the best accuracy followed by ev*GW*_0_*@*PBE with an MAE of 0.27 and 0.36 eV, respectively, which is consistent with the conclusion for the CORE65 set benchmark.

**Table 3:** Relative C 1s CLBEs for the ETFA Molecule^a^

	C1	C2	C3	C4	ME	MAE
experiment^103^	8.25	4.81	1.87	0.00		
experiment^102^	**7.46**	**4.33**	**1.72**	**0.00**		
ev*GW*_0_*@*PBE	–0.58	–0.42	–0.07	0.00	–0.36	0.36
*G*_0_*W*_0_*@*PBEh	0.40	0.38	0.14	0.00	0.31	0.31
*G*_Δ*H*_*W*_0_*@*PBE	–0.50	–0.42	–0.07	0.00	–0.33	0.33
ev*GW@*PBE	–0.47	–0.39	–0.05	0.00	–0.30	0.30
*G*_RS_*W*_RS_*@*PBE	–0.58	–0.44	–0.01	0.00	–0.34	0.34
*G*_RS_*W*_RS_*@*PBE0	–0.10	0.02	0.14	0.00	0.02	0.09
*G*_RS_*W*_RS_*@*B3LYP	–0.03	0.05	0.10	0.00	0.04	0.06
*G*_RS_*W*_RS_@PBEh	0.21	0.31	0.20	0.00	0.24	0.24
ev*GW*_0_*@*PBE^45^	–0.32	–0.09	0.05	0.00	–0.12	0.15
ΔSCAN^18^	–0.32	–0.25	–0.12	0.00	–0.23	0.23
ΔCCSD(T)^8^	–0.12	–0.01	–0.08	0.00	–0.07	0.07

a The deviation Δ_exp_ with respect to experiment and the corresponding ME and MAE are computed with the experimental data by Travnikova et al.^102^ (in bold), where Δ_exp_ = ΔCLBE_*i*_^theory^ – ΔCLBE_*i*_^exp^. The relative energies ΔCLBE are computed with respect to C4. The labels of the C atoms are given in the inset of Figure 6a. For the @PBEh calculations, we use α = 0.45.

Turning to the relative CLBEs, the three partial self-consistent schemes and *G*_0_*W*_0_*@*PBEh yield MAEs around 0.3 eV, which is only slightly worse than the CORE65 MAEs for C 1s. ev*GW*_0_, ev*GW*, and *G*_Δ*H*_*W*_0_ underestimate all shifts, while *G*_0_*W*_0_*@*PBEh overestimates the shifts of the more “descreened” C atoms (C1 and C2), see Figure 6b. *G*_RS_*W*_RS_ also provides good shifts for ETFA. The RS results are again not completely independent on the starting point. With PBE, the C 1s shifts are similarly underestimated as for the self-consistent schemes, while they are slightly too large with PBEh. The RS schemes with the conventional hybrid functionals PBE0 and B3LYP yield, with MAEs below 0.1 eV, the best overall result of the 8 investigated schemes. Furthermore, except for *G*_RS_*W*_RS_ with hybrid starting points, we find that our predictions are worse progressively with the electronegativity of the substituents at the C atoms. The relative CLBEs of C1 (CF_3_) and C2 (carbonyl) seem to be the ones that are more difficult to predict, while the predictions of the C3 (CH_2_) shifts are mostly within 0.1 eV of the experimental references. However, this trend can be also found in the deviations between the experimental references, see Table 3, that is, deviations between the experiment values increase with the “descreening” of the C atoms.

Finally, we compare our results to previously published computational XPS data.^8,18,45,102,104−106^ We focus here on the results from (i) the same method (ev*GW*_0_), (ii) one of the most successful functionals for ΔSCF (ΔSCAN), and (iii) from a higher-level method (coupled cluster), see Tables 2 and 3. All three literature results include relativistic effects. The previous ev*GW*_0_*@*PBE study^45^ uses the same correction scheme^50^ as employed in this work. The ΔSCAN results were obtained with the atomic zeroth-order regular approximation.^82^ For the delta coupled-cluster double results with triple correction (ΔCCSD(T)),^8^ scalar relativistic effects were taken into account by an exact two-component theory. The ΔSCAN and coupled-cluster results were computed for the anti-anti conformer, whereas the ev*GW*_0_ literature data are actually an average of the CLBEs of both conformers.

The absolute ev*GW*_0_*@*PBE CLBEs from ref (45) are less underestimated with respect to experiment in comparison with our results. The largest differences are observed for the descreened C1 and C2 atoms. The previously published ev*GW*_0_ results^45^ are not extrapolated but obtained with a basis set with more core functions. As already discussed for the CORE65 results, substantially increasing the amount of core functions seems to cure the (small) systematic underestimation of ev*GW*_0_ for absolute CLBEs. The predictions upon including more core functions should change on average by 0.25 eV for C atoms,^52^ which is close to the (maximal) 0.3 eV difference we observe here. However, similar to our results, the deviation from experiments is larger for C1/C2 than for C3/C4. Since adding the core functions seems to be more relevant for the descreened environments, the chemical shifts improve too, see Table 3. Some of the difference must be also attributed to the inclusion of the anti-gauche conformer in ref (45), which has slightly higher C 1s BEs.^102^

We note here that ref (45) contains also ETFA results with *G*_0_*W*_0_*@*PBEh(α = 0.45) employing core-rich basis sets. However, the α values were tuned with respect to experiment using an extrapolation scheme with the cc-pV*n*Z basis sets.^49^ An insufficiency in the basis set description (i.e., here a systematic underestimation) would be partly absorbed in the α value, which is the reason why our *G*_0_*W*_0_*@*PBEh results will agree better with experiment.

It has been recently shown that the SCAN functional yields excellent absolute and relative CLBEs for molecules^17^ and solids.^19^ We find that this is also true for the ETFA molecule. ΔSCAN yields the best MAE for absolute CLBEs, which is, however, very close to the ev*GW*_0_*@*PBE results^45^ with the core-function-rich basis set. For the relative CLBEs, the ΔSCAN is outperformed by partial self-consistent and RS *GW* approaches as well as coupled cluster.

The absolute ΔCCSD(T) core excitations^8^ are underestimated by 0.23–0.35 eV. However, these results were obtained at the cc-pVTZ level and are probably not fully converged.^28^ It is thus difficult to judge the performance of the method for absolute CLBEs. The chemical shifts on the contrary are often less affected by the basis set choice and ΔCCSD(T) yields together with *G*_RS_*W*_RS_(@PBE0 or @B3LYP) MAEs <0.1 eV.

### 4.4 Comparison of the Computational Cost

Comparing the computational cost of the five *GW* flavors employed in this work (*G*_0_*W*_0_, *G*_Δ*H*_*W*_0_, *G*_RS_*W*_RS_, ev*GW*_0_, and ev*GW*), the *G*_0_*W*_0_ scheme is the computationally least expensive one. For core-level calculations, highly accurate frequency techniques are required,^22^ such as the fully analytic evaluation of the self-energy via eq 6 or the contour deformation. This increases the computational cost compared to valence excitations, where schemes like the analytic continuation^31^ (AC) can be used. In conventional implementations, the AC scheme scales  with respect to system size *N* but fails to reproduce the self-energy structure for deep core states.^22^ The scaling of the fully analytic approach (eq 6) is  with respect to system size *N*. The complexity of the contour deformation technique increases for core levels from  (valence states) to  A detailed analysis of the scaling behavior of the contour deformation approach can be found in ref (22). For both techniques, fully analytic approach and contour deformation, the polarizability, which is the computational most expensive step in the *GW* calculation, is explicitly constructed in each iteration step of eq 1 (the QP equation). Typically, the QP equation converges within 10 steps; that is, the self-energy must be computed 10 times.

The computational cost of *G*_Δ*H*_*W*_0_*@*PBE is only slightly larger than for *G*_0_*W*_0_. The Hedin shift Δ*H* (see eq 12) is computed from  once before the iteration of eq 13. Given that the latter converges also in 10 steps, the self-energy needs to be calculated 11 instead of 10 times.

The *G*_RS_*W*_RS_ requires the subspace diagonalization of the RS Hamiltonian (eqs 21 and 22), which scales only , which is at least two orders of magnitudes lower than the frequency integration of the self-energy. In practice, the computational cost of a *G*_RS_*W*_RS_ calculation is only marginally larger than for a *G*_0_*W*_0_ calculation.

The computationally most expensive approaches discussed here are ev*GW*_0_ and ev*GW*. In ev*GW*_0_, the eigenvalues are iterated in *G* (outer loop), and in each step of the outer loop, we iterate the QP equation, that is, eq 1, not only for the core state of interest but for all states. Assuming again that the iteration converges within 10 steps, this implies that even for small molecules we evaluate the self-energy in the ev*GW*_0_ procedure several hundred times. ev*GW* is even more expensive because *W* is rebuilt in each step.

For core-level calculations, the eigenvalue self-consistent schemes are restricted to molecules with less than 20–25 atoms, while *G*_0_*W*_0_*@*PBEh, *G*_Δ*H*_*W*_0_, and *G*_RS_*W*_RS_ are equally applicable to larger systems. In our previous work,^24^ we computed CLBEs of structures of up to 110–120 atoms at the *G*_0_*W*_0_*@*PBEh level.

The comparison of the computational cost between *G*_0_*W*_0_*@*PBEh and ΔSCF depends on (i) the system size due to the different scalings of both methods and (ii) the choice of the functional for the ΔSCF calculations. A comprehensive assessment of the computational cost was given in our previous work,^24^ where we compared *G*_0_*W*_0_*@*PBEh to Δ Kohn–Sham^107^ (ΔKS) calculations with the PBE functional. (ΔKS is the projector augmented wave variant of the all-electron ΔSCF method.) We found that *G*_0_*W*_0_*@*PBEh is already ≈50 times more expensive for smaller molecules of around 20 atoms, while for isolated structures of ≈100 atoms the factor increases to 20,000. When using hybrid functionals for the ΔSCF calculation, the computational cost of *G*_0_*W*_0_*@*PBEh is similar to ΔSCF for small molecules since the evaluation of the exchange is the computational bottleneck in both cases. With increasing system size, the higher-scaling steps in *G*_0_*W*_0_ start to dominate. For structures with ≈100 atoms, we estimate that *G*_0_*W*_0_*@*PBEh is 1–2 orders of magnitude more expensive than hybrid-based ΔSCF calculations.

## 5 Conclusions

We have presented a benchmark study of different *GW* approaches for the prediction of absolute and relative CLBEs. In addition to the ev*GW*_0_*@*PBE and *G*_0_*W*_0_*@*PBEh(α = 0.45) methods, which were already investigated in ref (49), we have included ev*GW@*PBE and two new methods, namely, *G*_Δ*H*_*W*_0_ and *G*_RS_*W*_RS_, in our study. *G*_Δ*H*_*W*_0_ is an adaption of the “Hedin shift”^74,108^ to core levels and can be considered as computationally less expensive approximation to ev*GW*_0_. In the *G*_RS_*W*_RS_ approach, the RS Green’s function is used as a new starting point and, in contrast to our previous work,^67^ also used to compute the screened Coulomb interaction. The purpose of introducing the RS scheme is to reduce the dependence on the starting point, and the method has thus been tested with four different DFAs (PBE, PBE0, B3LYP, and PBEh (α = 0.45)).

By investigating the self-energy matrix elements and spectral functions, we have confirmed that ev*GW*_0_*@*PBE, *G*_0_*W*_0_*@*PBEh, *G*_Δ*H*_*W*_0_*@*PBE, *G*_RS_*W*_RS_*@*PBE, *G*_RS_*W*_RS_*@*PBE0, *G*_RS_*W*_RS_*@*B3LYP, and *G*_RS_*W*_RS_*@*PBEh yield a unique solution. *G*_0_*W*_0_ schemes starting from a GGA or hybrid DFT calculation with a low amount of exact exchange do not yield a distinct QP solution. A meaningful physical solution can thus not be obtained with standard approaches such as *G*_0_*W*_0_*@*PBE, *G*_0_*W*_0_*@*PBE0, and *G*_0_*W*_0_*@*B3LYP for CLBEs.

We have studied the CORE65 benchmark set and the C 1s excitations of the ETFA molecule with all 8 approaches, for which a physically reasonable solution behavior was confirmed. For the CORE65 set, ev*GW*_0_*@*PBE, *G*_0_*W*_0_*@*PBEh, and *G*_Δ*H*_*W*_0_*@*PBE yield with MAEs of 0.30, 0.33, and 0.25 eV, respectively, the best results. ev*GW* and *G*_RS_*W*_RS_ overestimate the absolute CLBEs by several electronvolts and are thus not suitable for the prediction of the absolute BEs. Nevertheless, the RS approaches significantly reduce the starting point dependence as intended. The relative CLBEs are reasonably reproduced with all methods, but in particular with the eigenvalue self-consistent schemes and *G*_Δ*H*_*W*_0_*@*PBE (MAEs < 0.2 eV). The methods exhibit a similar performance for the ETFA molecule, except that the RS approaches with standard hybrid functionals yield here the best chemical shifts.

The *G*_0_*W*_0_*@*PBEh(α) approach was introduced in our previous work^49^ as computationally affordable alternative to ev*GW*_0_ that can mimic to some extent the effect of eigenvalue self-consistency in *G*. However, the α-tuning is methodologically unsatisfying since the optimal α increases with the atomic number and an individual tuning for each element is in principle required and in fact mandatory for heavier elements. We therefore recommend to use the *G*_Δ*H*_*W*_0_*@*PBE approach instead, which is in terms of computational cost comparable to *G*_0_*W*_0_.

Finally, we found that ev*GW*_0_*@*PBE and *G*_Δ*H*_*W*_0_*@*PBE systematically underestimate the experiment. Our comparison to the ETFA literature results and very recent work^52^ suggest that this slight but systematic underestimation can be cured by very large, core-rich basis sets, which might improve the agreement with experiment even further. Future work will consider this and focus on the development of compact and computationally efficient NAO basis sets for core-level *GW* calculations.

## Supporting Information Available

CORE65 benchmark results, solution behavior of *G*_RS_*W*_0_, errors of KS-DFT for predicting absolute CLBEs and relative CLBEs, starting point dependence on the tuning parameter in PBEh, and geometry of ETFA (PDF)

## Notes

The data are available in the Supporting Information, and the input and output files of the FHI-aims calculations are available from the NOMAD database.^109,110^
