# Supplementary material for: Benchmark of GW Methods for Core-Level Binding Energies
Source: J Chem Theory Comput. 2022 Nov 2;18(12):7570–85. doi: 10.1021/acs.jctc.2c00617 (PMC9753590; doi:10.1021/acs.jctc.2c00617)
Supplement: Supplementary file 1 — ct2c00617_si_001.pdf [file ct2c00617_si_001.pdf]

Supporting Information: Benchmark of GW Methods for Core-Level Binding Energies

Jiachen Li,† Ye Jin,† Patrick Rinke,‡ Weitao Yang,† and Dorothea Golze*,¶,‡

†*Department of Chemistry, Duke University, Durham, NC 27708, USA*

‡*Department of Applied Physics, Aalto University, Otakaari 1, FI-02150 Espoo, Finland*

¶*Faculty of Chemistry and Food Chemistry, Technische Universität Dresden, 01062 Dresden, Germany*

E-mail: dorothea.golze@tu-dresden.de

1 CORE65 results with evGW, $G_{\Delta\mathrm{H}}W_0$ and $G_{RS}W_{RS}$

The labels for the carbon atoms of nitrobenzene and phenylacetylene are used as defined in the SI of Ref. 1. The experimental values are taken from the SI of Ref. 1, where also the citation for each experimental value is listed. The core-level BEs in Table S1 and S2 include the relativistic corrections used in Refs. 1,2.

Table S1: Core-level binding energies (BEs) as obtained from evGW@PBE and $G_{\Delta\mathrm{H}}W_0$@PBE. All values in eV. The BEs have been extrapolated to the complete basis set limit by performing a linear regression with respect to the inverse of the total number of basis functions using the cc-pVnZ basis set series ($n = 3-6$). Relativistic effects are taken into account for both methods.

CAS	name	formula	core level	evGW@PBE	$G_{\Delta\mathrm{H}}W_0$@PBE	Exp.
74-82-8	methane	CH_4	C1s	292.22	290.65	290.84
74-84-0	ethane	C_2H_6	C1s	292.09	290.75	290.71
74-85-1	ethene	C_2H_4	C1s	292.44	290.85	290.82
74-86-2	ethyne	C_2H_2	C1s	292.89	291.22	291.25
630-08-0	carbon monoxide	CO	O1s	544.19	542.14	542.1
630-08-0	carbon monoxide	CO	C1s	297.38	295.84	296.23
124-38-9	carbon dioxide	CO_2	O1s	543.05	540.85	541.32
124-38-9	carbon dioxide	CO_2	C1s	298.64	297.26	297.70
75-73-0	tetrafluoromethane	CF_4	F1s	696.75	694.52	695.2
75-73-0	tetrafluoromethane	CF_4	C1s	302.74	301.25	301.90
593-53-3	fluoromethane	CH_3F	F1s	694.04	691.97	692.4
593-53-3	fluoromethane	CH_3F	C1s	295.04	293.54	293.56
75-46-7	trifluoromethane	CHF_3	F1s	695.87	693.59	694.1

Table S1: Continued

CAS	name	formula	core level	evGW@PBE	$G_{\Delta H}W_0$@PBE	Exp.
75-46-7	trifluoromethane	CHF_3	C1s	300.32	298.77	299.16
67-56-1	methanol	CH_3OH	O1s	540.59	538.65	538.88
67-56-1	methanol	CH_3OH	C1s	293.91	292.43	292.3
50-00-0	formaldehyde	CH_2O	O1s	541.03	539.04	539.33
50-00-0	formaldehyde	CH_2O	C1s	296.01	294.52	294.38
115-10-6	dimethyl ether	CH_3OCH_3	O1s	539.99	538.27	538.36
115-10-6	dimethyl ether	CH_3OCH_3	C1s	293.65	292.19	292.17
64-18-6	formic acid	HCOOH	O1s (OH)	542.19	540.25	540.69
64-18-6	formic acid	HCOOH	O1s (C=O)	540.52	538.51	539.02
64-18-6	formic acid	HCOOH	C1s	296.96	295.46	295.75
67-64-1	acetone	$(CH_3)_2CO$	O1s	539.41	537.53	537.73
67-64-1	acetone	$(CH_3)_2CO$	C1s (C=O)	295.01	293.62	293.88
67-64-1	acetone	$(CH_3)_2CO$	C1s (CH_3)	292.58	291.09	291.23
107-31-3	methyl formate	HCO_2CH_3	O1s (OCH_3)	541.48	539.58	539.64
107-31-3	methyl formate	HCO_2CH_3	O1s (C=O)	540.06	538.00	538.24
64-19-7	acetic acid	CH_3COOH	O1s (OH)	541.57	539.73	540.10
64-19-7	acetic acid	CH_3COOH	O1s (C=O)	539.78	537.76	538.31
64-19-7	acetic acid	CH_3COOH	C1s (COOH)	296.48	295.01	295.35
64-19-7	acetic acid	CH_3COOH	C1s (CH_3)	293.00	291.51	291.55
7732-18-5	water	H_2O	O1s	541.43	539.45	539.7
10028-15-	ozone	O_3	O1s middle	548.77	546.80	546.44
10028-15-	ozone	O_3	O1s terminal	543.45	541.23	541.75
7782-44-7	oxygen	O_2	O1s weaker	546.12	543.76	544.2
7782-44-7	oxygen	O_2	O1s stronger	545.02	542.88	543.1
7727-37-9	nitrogen	N_2	N1s	411.42	409.87	409.93
7664-41-7	ammonia	NH_3	N1s	407.21	405.53	405.52
74-90-8	hydrogen cyanide	HCN	N1s	408.54	406.68	406.8
74-90-8	hydrogen cyanide	HCN	C1s	294.81	293.34	293.5
75-05-8	acetonitrile	CH_3CN	N1s	407.25	405.38	405.58
75-05-8	acetonitrile	CH_3CN	C1s (CH_3)	294.12	292.56	292.88
75-05-8	acetonitrile	CH_3CN	C1s (CN)	294.03	292.36	292.60
56-40-6	glycine	$C_2H_5NO_2$	O1s (OH)	541.68	539.71	540.2
56-40-6	glycine	$C_2H_5NO_2$	O1s (C=O)	539.92	537.89	538.4
56-40-6	glycine	$C_2H_5NO_2$	N1s	407.06	405.20	405.4
56-40-6	glycine	$C_2H_5NO_2$	C1s (COOH)	296.30	294.83	295.2
56-40-6	glycine	$C_2H_5NO_2$	C1s (CH_2)	293.70	292.21	292.3
110-86-1	pyridine	C_5H_5N	N1s	406.31	404.66	404.82
109-97-7	pyrrole	C_4H_4NH	N1s	407.83	406.25	406.18
62-53-3	aniline	$C_6H_5NH_2$	N1s	406.85	405.09	405.31
57-13-6	urea	$CO(NH_2)_2$	O1s	538.67	536.67	537.19
57-13-6	urea	$CO(NH_2)_2$	N1s	407.69	405.94	406.09
57-13-6	urea	$CO(NH_2)_2$	C1s	295.98	294.49	294.84
74-89-5	methylamine	CH_3NH_2	N1s	406.59	404.94	405.17
98-95-3	nitrobenzene	$C_6H_5NO_2$	O1s	540.17	538.18	538.63
98-95-3	nitrobenzene	$C_6H_5NO_2$	N1s	413.01	411.48	411.6
98-95-3	nitrobenzene	$C_6H_5NO_2$	C1s (C1)	293.39	291.98	292.08
98-95-3	nitrobenzene	$C_6H_5NO_2$	C1s (C2-4)	292.54	291.08	291.13
71-43-2	benzene	C_6H_6	C1s	291.85	290.38	290.38
536-74-3	phenylacetylene	C_8H_6	C1s (C3)	292.49	291.05	290.88
536-74-3	phenylacetylene	C_8H_6	C1s (C2)	292.05	290.57	290.55
536-74-3	phenylacetylene	C_8H_6	C1s (C4-6)	291.93	290.39	290.16
536-74-3	phenylacetylene	C_8H_6	C1s (C1)	291.67	290.04	289.75

Table S2: Core-level binding energies as obtained from $G_{RS}W_{RS}$@PBE, $G_{RS}W_{RS}$@PBE0, $G_{RS}W_{RS}$@B3LYP and $G_{RS}W_{RS}$@PBEh(α=0.45). All values in eV. The BEs have been extrapolated to the complete basis set limit by performing a linear regression with respect to the inverse of the total number of basis functions using the cc-pV3Z and cc-pV4Z basis set sets. Relativistic effects are taken into account for all methods.

name	formula	core level	$G_{RS}W_{RS}$@PBE	$G_{RS}W_{RS}$@PBE0	$G_{RS}W_{RS}$@B3LYP	$G_{RS}W_{RS}$@PBEh(α=0.45)
methane	CH_4	C1s	294.73	295.11	294.85	295.50
ethane	C_2H_6	C1s	294.68	295.10	294.77	295.48
ethene	C_2H_4	C1s	294.98	295.43	295.10	295.90
ethyne	C_2H_2	C1s	295.34	563.88	295.49	296.19
carbon monoxide	CO	O1s	547.78	548.01	547.53	548.26
carbon monoxide	CO	C1s	299.46	300.00	299.76	300.61
carbon dioxide	CO_2	O1s	546.78	547.17	546.65	547.59
carbon dioxide	CO_2	C1s	300.73	301.68	301.50	302.67
tetrafluoromethane	CF_4	F1s	701.48	701.71	701.24	701.75
tetrafluoromethane	CF_4	C1s	305.12	305.94	305.82	306.82
fluoromethane	CH_3F	F1s	699.06	699.15	698.70	699.28
fluoromethane	CH_3F	C1s	297.53	297.94	297.74	298.44
trifluoromethane	CHF_3	F1s	700.85	701.09	700.73	701.30
trifluoromethane	CHF_3	C1s	302.61	303.45	303.35	304.18
methanol	CH_3OH	O1s	544.63	544.85	544.34	545.12
methanol	CH_3OH	C1s	296.36	296.79	296.56	297.30
formaldehyde	CH_2O	O1s	545.13	545.36	544.87	545.59
formaldehyde	CH_2O	C1s	298.37	298.85	298.65	299.40
dimethyl ether	CH_3OCH_3	O1s	544.48	544.67	544.09	544.91
dimethyl ether	CH_3OCH_3	C1s	296.26	296.73	296.49	297.23
formic acid	HCOOH	O1s (OH)	546.35	546.62	546.12	546.93
formic acid	HCOOH	O1s (C=O)	544.70	544.94	544.46	545.22
formic acid	HCOOH	C1s	299.33	300.02	299.82	300.76
acetone	$(CH_3)_2CO$	O1s	544.26	544.61	544.05	544.93
acetone	$(CH_3)_2CO$	C1s (C=O)	297.62	298.19	297.93	298.80
acetone	$(CH_3)_2CO$	C1s (CH_3)	295.25	295.68	295.44	296.13
methyl formate	HCO_2CH_3	O1s (OCH_3)	545.81	546.10	545.52	546.38
methyl formate	HCO_2CH_3	O1s (C=O)	544.29	544.62	544.11	544.92
acetic acid	CH_3COOH	O1s (OH)	545.84	546.17	545.66	546.50
acetic acid	CH_3COOH	O1s (C=O)	544.11	544.46	543.94	544.77
acetic acid	CH_3COOH	C1s (COOH)	298.92	299.66	299.44	300.43
acetic acid	CH_3COOH	C1s (CH_3)	295.65	296.09	295.83	296.55
water	H_2O	O1s	545.05	545.27	544.85	545.53
ozone	O_3	O1s middle	552.57	553.30	552.71	554.17
ozone	O_3	O1s terminal	547.58	548.08	547.62	548.63
oxygen	O_2	O1s weaker	†	†	†	†
oxygen	O_2	O1s stronger	†	†	†	†
nitrogen	N_2	N1s	413.99	414.20	414.07	414.88
ammonia	NH_3	N1s	410.45	410.54	410.39	411.14
hydrogen cyanide	HCN	N1s	411.68	411.88	411.68	412.58
hydrogen cyanide	HCN	C1s	297.10	297.36	297.43	297.91
acetonitrile	CH_3CN	N1s	410.92	411.53	410.91	411.58
acetonitrile	CH_3CN	C1s (CH_3)	296.68	297.02	296.84	297.54
acetonitrile	CH_3CN	C1s (CN)	296.37	296.82	296.53	297.48
glycine	$C_2H_5NO_2$	O1s (OH)	545.97	546.20	545.70	546.55
glycine	$C_2H_5NO_2$	O1s (C=O)	545.02	545.41	544.67	543.92
glycine	$C_2H_5NO_2$	N1s	410.64	410.98	410.51	411.38
glycine	$C_2H_5NO_2$	C1s (COOH)	298.92	299.58	299.33	300.29
glycine	$C_2H_5NO_2$	C1s (CH_2)	296.49	296.77	296.67	297.37

Table S2: Continued

name	formula	core level	$G_{RS}W_{RS}$@PBE	$G_{RS}W_{RS}$@PBE0	$G_{RS}W_{RS}$@B3LYP	$G_{RS}W_{RS}$@PBEh(α=0.45)
pyridine	C_5H_5N	N1s	410.36	410.81	410.29	411.31
pyrrole	C_4H_4NH	N1s	411.60	411.94	411.43	412.42
aniline	$C_6H_5NH_2$	N1s	410.64	410.97	410.50	411.23
urea	$CO(NH_2)_2$	O1s	543.03	543.32	542.80	543.60
urea	$CO(NH_2)_2$	N1s	411.18	411.62	411.25	412.05
urea	$CO(NH_2)_2$	C1s	298.48	299.22	299.00	300.05
methylamine	CH_3NH_2	N1s	410.25	410.55	410.10	410.93
nitrobenzene	$C_6H_5NO_2$	O1s	544.96	545.40	544.85	545.69
nitrobenzene	$C_6H_5NO_2$	N1s	416.71	417.71	417.14	418.39
nitrobenzene	$C_6H_5NO_2$	C1s (C1)	296.49	297.02	296.65	297.45
nitrobenzene	$C_6H_5NO_2$	C1s (C2-4)	295.59	296.10	295.74	296.51
benzene	C_6H_6	C1s	294.73	295.28	294.84	295.56
phenylacetylene	C_8H_6	C1s (C3)	295.48	295.95	295.58	296.29
phenylacetylene	C_8H_6	C1s (C2)	294.94	295.42	295.06	295.74
phenylacetylene	C_8H_6	C1s (C4-6)	294.93	295.41	295.04	295.78
phenylacetylene	C_8H_6	C1s (C1)	294.86	295.48	294.98	295.51

2 ev*GW* results without relativistic correction

Table S3: Non-relativistic core-level binding energies from ev*GW*@PBE using the cc-pV*n*Z basis set series with $n = 3 - 6$. Extrapolated values (∞),standard error (SE) and correlation coefficient R^2. The last column indicates the basis sets used for the extrapolation; for most cases all data points ($n = 3 - 6$) are included; see SI of Ref. 1 for a discussion why $n = 5, 6$ are sometimes excluded. All values in eV.

				ev*GW*@PBE							
CAS	name	formula	core level	$n = 3$	$n = 4$	$n = 5$	$n = 6$	∞	SE	R^2	n used
74-82-8	methane	CH_4	C1s	291.59	291.83	291.90	292.08	292.11	0.94	0.05	3-6
74-84-0	ethane	C_2H_6	C1s	291.42	291.68	291.75	291.94	291.97	0.93	0.06	3-6
74-85-1	ethene	C_2H_4	C1s	291.72	291.99	292.08	292.27	292.32	0.96	0.06	3-6
74-86-2	ethyne	C_2H_2	C1s	292.18	292.40	292.53	292.74	292.77	0.92	0.08	3-6
630-08-0	carbon monoxide	CO	O1s	543.56	543.64	543.61	543.73	543.77	0.98	0.02	3,4,6
630-08-0	carbon monoxide	CO	C1s	296.79	297.00	297.04	297.21	297.26	0.92	0.06	3-6
124-38-9	carbon dioxide	CO_2	O1s	542.25	542.36	542.37	542.57	542.62	0.94	0.07	3,4,6
124-38-9	carbon dioxide	CO_2	C1s	297.82	298.10	298.22	298.43	298.52	0.95	0.07	3-6
75-73-0	tetrafluoromethane	CF_4	F1s	696.05	696.05	695.90	695.96	696.04			3,4
75-73-0	tetrafluoromethane	CF_4	C1s	301.90	302.29	302.32	302.50	302.62	0.96	0.07	3-6
593-53-3	fluoromethane	CH_3F	F1s	693.34	693.34	693.26	693.32	693.33			3,4
593-53-3	fluoromethane	CH_3F	C1s	294.25	294.55	294.64	294.87	294.92	0.94	0.07	3-6
75-46-7	trifluoromethane	CHF_3	F1s	695.16	695.16	695.08	695.12	695.16			3,4
75-46-7	trifluoromethane	CHF_3	C1s	299.39	299.78	299.88	300.08	300.20	0.98	0.05	3-6
67-56-1	methanol	CH_3OH	O1s	539.86	539.97	539.97	540.13	540.17	0.94	0.05	3,4,6
67-56-1	methanol	CH_3OH	C1s	293.14	293.43	293.53	293.74	293.79	0.95	0.07	3-6
50-00-0	formaldehyde	CH_2O	O1s	540.49	540.56	540.57	540.67	540.61	0.98	0.01	3-5
50-00-0	formaldehyde	CH_2O	C1s	295.22	295.53	295.61	295.82	295.89	0.95	0.06	3-6
115-10-6	dimethyl ether	CH_3OCH_3	O1s	539.42	539.49	539.53	539.62	539.57	1.00	0.00	3-5
115-10-6	dimethyl ether	CH_3OCH_3	C1s	292.90	293.19	293.28	293.48	293.53	0.94	0.06	3-6

† spin-polarized $G_{RS}W_{RS}$ calculations not available in QM4D

Table S3: Continued

CAS	name	formula	core level	ev*GW*@PBE $n = 3$	$n = 4$	$n = 5$	$n = 6$	∞	SE	R^2	n used
64-18-6	formic acid	HCOOH	O1s (OH)	541.55	541.66	541.69	541.84	541.76	1.00	0.01	3-5
64-18-6	formic acid	HCOOH	O1s (C=O)	539.80	539.91	539.96	540.08	540.10	0.90	0.04	3-6
64-18-6	formic acid	HCOOH	C1s	296.09	296.44	296.54	296.75	296.84	0.96	0.06	3-6
67-64-1	acetone	$(CH_3)_2CO$	O1s	538.77	538.88	538.92	539.03	538.99	1.00	0.01	3-5
67-64-1	acetone	$(CH_3)_2CO$	C1s (C=O)	294.26	294.54	294.64	294.84	294.89	0.95	0.06	3-6
67-64-1	acetone	$(CH_3)_2CO$	C1s (CH_3)	291.84	292.12	292.21	292.42	292.46	0.94	0.07	3-6
107-31-3	methyl formate	HCO_2CH_3	O1s (OCH_3)	540.83	540.92	540.99	541.11	541.05	0.98	0.02	3-5
107-31-3	methyl formate	HCO_2CH_3	O1s (C=O)	539.31	539.43	539.50	539.62	539.64	0.90	0.05	3-6
64-19-7	acetic acid	CH_3COOH	O1s (OH)	540.89	541.02	541.06	541.20	541.15	0.99	0.01	3-5
64-19-7	acetic acid	CH_3COOH	O1s (C=O)	539.01	539.15	539.22	539.34	539.36	0.92	0.04	3-6
64-19-7	acetic acid	CH_3COOH	C1s (COOH)	295.61	295.96	296.07	296.27	296.36	0.97	0.06	3-6
64-19-7	acetic acid	CH_3COOH	C1s (CH_3)	292.23	292.53	292.61	292.83	292.88	0.94	0.07	3-6
7732-18-5	water	H_2O	O1s	540.67	540.80	540.79	540.96	541.00	0.98	0.04	3,4,6
10028-15-6	ozone	O_3	O1s middle	548.05	548.16	548.18	548.29	548.35	0.98	0.03	3,4,6
10028-15-6	ozone	O_3	O1s terminal	542.92	542.97	542.97	543.07	543.03			3,4
7782-44-7	oxygen	O_2	O1s weaker	545.61	545.65	545.55	545.68	545.70	1.00	0.00	3,4,6
7782-44-7	oxygen	O_2	O1s stronger	544.48	544.53	544.49	544.60	544.59			3,4
7727-37-9	nitrogen	N_2	N1s	410.75	410.96	410.99	411.12	411.18	0.95	0.04	3-6
7664-41-7	ammonia	NH_3	N1s	406.32	406.66	406.75	406.89	406.98	0.99	0.03	3-6
74-90-8	hydrogen cyanide	HCN	N1s	407.76	408.02	408.06	408.26	408.31	0.92	0.07	3-6
74-90-8	hydrogen cyanide	HCN	C1s	294.13	294.35	294.45	294.64	294.69	0.93	0.07	3-6
75-05-8	acetonitrile	CH_3CN	N1s	406.48	406.74	406.80	406.95	407.02	0.96	0.05	3-6
75-05-8	acetonitrile	CH_3CN	C1s (CH_3)	293.40	293.67	293.75	293.96	294.01	0.93	0.07	3-6
75-05-8	acetonitrile	CH_3CN	C1s (CN)	293.31	293.56	293.66	293.87	293.91	0.93	0.07	3-6
56-40-6	glycine	$C_2H_5NO_2$	O1s (OH)	540.88	541.03	541.09	541.24	541.25	0.90	0.05	3-6
56-40-6	glycine	$C_2H_5NO_2$	O1s (C=O)	539.10	539.24	539.34	539.47	539.50	0.93	0.05	3-6
56-40-6	glycine	$C_2H_5NO_2$	N1s	406.18	406.47	406.55	406.77	406.82	0.93	0.07	3-6
56-40-6	glycine	$C_2H_5NO_2$	C1s (COOH)	295.39	295.75	295.88	296.09	296.18	0.97	0.06	3-6
56-40-6	glycine	$C_2H_5NO_2$	C1s (CH_2)	292.84	293.18	293.28	293.50	293.58	0.96	0.07	3-6
110-86-1	pyridine	C_5H_5N	N1s	405.58	405.76	405.88	406.03	406.07	0.94	0.06	3-6
109-97-7	pyrrole	C_4H_4NH	N1s	406.98	407.22	407.36	407.55	407.60	0.94	0.07	3-6
62-53-3	aniline	$C_6H_5NH_2$	N1s	405.96	406.21	406.37	406.56	406.62	0.94	0.07	3-6
57-13-6	urea	$CO(NH_2)_2$	O1s	537.86	538.01	538.08	538.22	538.24	0.92	0.05	3-6
57-13-6	urea	$CO(NH_2)_2$	N1s	406.76	407.08	407.18	407.38	407.46	0.96	0.06	3-6
57-13-6	urea	$CO(NH_2)_2$	C1s	295.06	295.42	295.55	295.77	295.86	0.97	0.06	3-6
74-89-5	methylamine	CH_3NH_2	N1s	406.04	406.19	406.26	406.44	406.35	1.00	0.00	3-5
98-95-3	nitrobenzene	$C_6H_5NO_2$	O1s	539.46	539.60	539.65	*	539.74	0.99	0.01	3-5
98-95-3	nitrobenzene	$C_6H_5NO_2$	N1s	412.15	412.44	412.58	*	412.78	1.00	0.00	3-5
98-95-3	nitrobenzene	$C_6H_5NO_2$	C1s (C1)	292.67	292.94	293.09	*	293.27	1.00	0.02	3-5
98-95-3	nitrobenzene	$C_6H_5NO_2$	C1s (C2-4)	291.82	292.10	292.24	*	292.42	1.00	0.01	3-5
71-43-2	benzene	C_6H_6	C1s	291.09	291.33	291.48	291.67	291.73	0.94	0.07	3-6
536-74-3	phenylacetylene	C_8H_6	C1s (C3)	291.76	292.02	292.12	292.31	292.37	0.94	0.06	3-6
536-74-3	phenylacetylene	C_8H_6	C1s (C2)	291.34	291.59	291.67	291.89	291.93	0.92	0.08	3-6
536-74-3	phenylacetylene	C_8H_6	C1s (C4-6)	291.20	291.47	291.56	291.76	291.82	0.95	0.06	3-6
536-74-3	phenylacetylene	C_8H_6	C1s (C1)	290.94	291.24	291.27	291.51	291.55	0.91	0.08	3-6

* ev*GW* outer cycle not converged

3 $G_{\Delta H}W_0$ results without relativistic correction

Table S4: Non-relativistic core-level binding energies from $G_{\Delta H}W_0$@PBE using the cc-pVnZ basis set series with $n = 3 - 6$. Extrapolated values (∞), standard error (SE) and correlation coefficient R^2. The last column indicates the basis sets used for the extrapolation; for most cases all data points ($n = 3-6$) are included; see SI of Ref. 1 for a discussion why $n = 5, 6$ are sometimes excluded. All values in eV.

				$G_{\Delta H}W_0$@PBE							
CAS	name	formula	core level	$n = 3$	$n = 4$	$n = 5$	$n = 6$	∞	SE	R^2	n used
74-82-8	methane	CH_4	C1s	290.00	290.27	290.39	290.44	290.53	1.00	0.00	3-6
74-84-0	ethane	C_2H_6	C1s	289.89	290.20	290.25	290.53	290.63	0.98	0.07	3,4,6
74-85-1	ethene	C_2H_4	C1s	290.10	290.41	290.45	290.71	290.74	0.90	0.09	3-6
74-86-2	ethyne	C_2H_2	C1s	290.50	290.76	290.93	291.21	291.10	0.99	0.04	3-5
630-08-0	carbon monoxide	CO	O1s	541.44	541.53	541.41	541.67	541.72	0.97	0.04	3,4,6
630-08-0	carbon monoxide	CO	C1s	295.15	295.38	295.48	295.65	295.72	0.95	0.06	3-6
124-38-9	carbon dioxide	CO_2	O1s	540.15	540.28	540.33	540.51	540.42	1.00	0.01	3-5
124-38-9	carbon dioxide	CO_2	C1s	296.26	296.22	296.85	296.96	297.14	1.00	0.00	3,5,6
75-73-0	tetrafluoromethane	CF_4	F1s	693.61	693.64	693.62	693.79	693.81	0.81	0.07	3,4,6
75-73-0	tetrafluoromethane	CF_4	C1s	300.16	300.63	300.75	300.97	301.13	0.98	0.06	3-6
593-53-3	fluoromethane	CH_3F	F1s	691.23	691.24	691.20	691.21	691.26			3,4
593-53-3	fluoromethane	CH_3F	C1s	292.57	292.99	293.08	293.34	293.42	0.95	0.08	3-6
75-46-7	trifluoromethane	CHF_3	F1s	692.80	692.84	692.81	692.91	692.88			3,4
75-46-7	trifluoromethane	CHF_3	C1s	297.69	298.15	298.26	298.51	298.65	0.97	0.07	3-6
67-56-1	methanol	CH_3OH	O1s	537.96	538.09	538.06	538.41	538.22			3,4
67-56-1	methanol	CH_3OH	C1s	291.53	291.87	291.99	292.27	292.32	0.93	0.09	3-6
50-00-0	formaldehyde	CH_2O	O1s	538.36	538.48	538.35	538.73	538.61			3,4
50-00-0	formaldehyde	CH_2O	C1s	293.60	293.95	294.06	294.34	294.40	0.93	0.09	3-6
115-10-6	dimethyl ether	CH_3OCH_3	O1s	537.51	537.61	537.72	537.83	537.84	0.90	0.05	3-6
115-10-6	dimethyl ether	CH_3OCH_3	C1s	291.32	291.66	291.77	292.03	292.08	0.94	0.08	3-6
64-18-6	formic acid	HCOOH	O1s (OH)	539.47	539.62	539.66	539.80	539.82	0.91	0.05	3-6
64-18-6	formic acid	HCOOH	O1s (C=O)	537.65	537.80	537.63	538.02	538.08	0.95	0.07	3,4,6
64-18-6	formic acid	HCOOH	C1s	294.50	294.89	295.01	295.24	295.34	0.97	0.07	3-6
67-64-1	acetone	$(CH_3)_2CO$	O1s	536.69	536.83	536.67	537.05	537.10	0.93	0.08	3,4,6
67-64-1	acetone	$(CH_3)_2CO$	C1s (C=O)	292.55	293.03	293.17	293.37	293.50	0.99	0.05	3-6
67-64-1	acetone	$(CH_3)_2CO$	C1s (CH_3)	290.27	290.60	290.67	290.92	290.97	0.93	0.08	3-6
107-31-3	methyl formate	HCO_2CH_3	O1s (OCH_3)	538.79	538.92	539.04	539.12	539.16	0.95	0.03	3-6
107-31-3	methyl formate	HCO_2CH_3	O1s (C=O)	537.19	537.34	537.29	537.52	537.58	0.97	0.04	3,4,6
64-19-7	acetic acid	CH_3COOH	O1s (OH)	538.87	539.03	539.00	539.25	539.31	0.96	0.06	3,4,6
64-19-7	acetic acid	CH_3COOH	O1s (C=O)	536.93	537.08	536.99	537.28	537.34	0.97	0.05	3,4,6
64-19-7	acetic acid	CH_3COOH	C1s (COOH)	294.05	294.42	294.58	294.80	294.89	0.97	0.06	3-6
64-19-7	acetic acid	CH_3COOH	C1s (CH_3)	290.65	290.98	291.08	291.33	291.39	0.94	0.08	3-6
7732-18-5	water	H_2O	O1s	538.68	538.83	538.75	538.97	539.02	0.99	0.02	3,4,6
10028-15-6	ozone	O_3	O1s middle	545.91	546.04	546.01	546.30	546.37	0.93	0.09	3,4,6
10028-15-6	ozone	O_3	O1s terminal	540.63	540.71	540.75	540.88	540.80	1.00	0.00	3-5
7782-44-7	oxygen	O_2	O1s weaker	543.18	543.23	543.30	543.37	543.34	0.91	0.04	3-5
7782-44-7	oxygen	O_2	O1s stronger	542.28	542.33	542.41	542.50	542.45	0.92	0.04	3-5
7727-37-9	nitrogen	N_2	N1s	408.89	408.87	409.13	409.36	409.64	0.98	0.08	4,5,6
7664-41-7	ammonia	NH_3	N1s	404.47	404.98	405.02	405.15	405.30	0.96	0.07	3-6
74-90-8	hydrogen cyanide	HCN	N1s	405.85	406.10	406.12	406.34	406.45	0.99	0.04	3,4,6
74-90-8	hydrogen cyanide	HCN	C1s	292.55	292.78	293.00	293.12	293.22	0.97	0.06	3-6
75-05-8	acetonitrile	CH_3CN	N1s	404.57	404.84	404.90	405.08	405.14	0.95	0.05	3-6
75-05-8	acetonitrile	CH_3CN	C1s (CH_3)	291.82	292.16	292.23	292.60	292.44	0.98	0.06	3-5
75-05-8	acetonitrile	CH_3CN	C1s (CN)	291.77	291.97	292.12	292.36	292.25	0.99	0.04	3-5

Table S4: Continued

				$G_{\Delta H}W_0$@PBE							
CAS	name	formula	core level	$n=3$	$n=4$	$n=5$	$n=6$	∞	SE	R^2	n used
56-40-6	glycine	$C_2H_5NO_2$	O1s (OH)	538.88	539.05	539.12	539.26	539.29	0.93	0.05	3-6
56-40-6	glycine	$C_2H_5NO_2$	O1s (C=O)	537.00	537.16	537.35	537.37	537.46	0.95	0.04	3-6
56-40-6	glycine	$C_2H_5NO_2$	N1s	404.62	404.77	404.87	405.07	404.96	0.99	0.02	3-5
56-40-6	glycine	$C_2H_5NO_2$	C1s (COOH)	293.80	294.21	294.38	294.59	294.71	0.98	0.05	3-6
56-40-6	glycine	$C_2H_5NO_2$	C1s (CH_2)	291.29	291.63	291.77	292.02	292.09	0.95	0.08	3-6
110-86-1	pyridine	C_5H_5N	N1s	403.89	404.06	404.22	404.39	404.43	0.91	0.08	3-6
109-97-7	pyrrole	C_4H_4NH	N1s	404.85	405.54	405.59	405.80	406.01	0.96	0.10	3-6
62-53-3	aniline	$C_6H_5NH_2$	N1s	404.30	404.53	404.61	404.83	404.86	0.91	0.08	3-6
57-13-6	urea	$CO(NH_2)_2$	O1s	535.77	535.95	535.78	536.18	536.25	0.96	0.06	3,4,6
57-13-6	urea	$CO(NH_2)_2$	N1s	405.02	405.31	405.46	405.61	405.70	0.98	0.04	3-6
57-13-6	urea	$CO(NH_2)_2$	C1s	293.61	293.94	294.07	294.31	294.37	0.95	0.08	3-6
74-89-5	methylamine	CH_3NH_2	N1s	404.35	404.52	404.61	404.80	404.70	1.00	0.01	3-5
98-95-3	nitrobenzene	$C_6H_5NO_2$	O1s	537.34	537.48	537.61	537.71	537.76	0.94	0.04	3-6
98-95-3	nitrobenzene	$C_6H_5NO_2$	N1s	410.36	410.79	410.74	411.06	411.24	1.00	0.01	3,4,6
98-95-3	nitrobenzene	$C_6H_5NO_2$	C1s (C1)	291.16	291.41	291.57	291.81	291.86	0.93	0.09	3-6
98-95-3	nitrobenzene	$C_6H_5NO_2$	C1s (C2-4)	290.23	290.51	290.67	290.89	290.97	0.95	0.07	3-6
71-43-2	benzene	C_6H_6	C1s	289.49	289.81	289.97	290.18	290.26	0.96	0.07	3-6
536-74-3	phenylacetylene	C_8H_6	C1s (C3)	290.18	290.51	290.62	290.87	290.94	0.94	0.08	3-6
536-74-3	phenylacetylene	C_8H_6	C1s (C2)	289.71	290.02	290.15	290.39	290.46	0.94	0.08	3-6
536-74-3	phenylacetylene	C_8H_6	C1s (C4-6)	289.54	289.84	289.98	290.21	290.28	0.94	0.08	3-6
536-74-3	phenylacetylene	C_8H_6	C1s (C1)	289.19	289.46	289.50	289.81	289.92	0.97	0.09	3,4,6

4 $G_{RS}W_{RS}$ results without relativistic correction

Table S5: Non-relativistic core-level binding energies from $G_{RS}W_{RS}$@PBE and $G_{RS}W_{RS}$@PBE0 using the cc-pVnZ basis set series with $n = 3, 4$ and 2-point extrapolated values (∞). For $G_{RS}W_{RS}$ calculations, the cc-pVnZ basis sets are treated as Cartesian Gaussian functions. All values in eV.

				$G_{RS}W_{RS}$@PBE			$G_{RS}W_{RS}$@PBE0		
CAS	name	formula	core level	$n=3$	$n=4$	∞	$n=3$	$n=4$	∞
74-82-8	methane	CH_4	C1s	293.74	294.22	294.62	294.06	294.57	294.99
74-84-0	ethane	C_2H_6	C1s	293.82	294.23	294.57	294.14	294.60	294.98
74-85-1	ethene	C_2H_4	C1s	294.25	294.58	294.86	294.55	294.96	295.31
74-86-2	ethyne	C_2H_2	C1s	294.50	294.88	295.22	294.88	295.30	295.69
630-08-0	carbon monoxide	CO	O1s	547.06	547.21	547.35	547.24	547.42	547.59
630-08-0	carbon monoxide	CO	C1s	298.41	298.88	299.35	298.89	299.39	299.88
124-38-9	carbon dioxide	CO_2	O1s	546.13	546.24	546.36	546.43	546.59	546.75
124-38-9	carbon dioxide	CO_2	C1s	299.60	300.11	300.61	300.39	300.98	301.56
75-73-0	tetrafluoromethane	CF_4	F1s	699.56	700.17	700.77	699.72	700.36	701.00
75-73-0	tetrafluoromethane	CF_4	C1s	303.68	304.34	305.01	304.54	305.18	305.82
593-53-3	fluoromethane	CH_3F	F1s	696.91	697.68	698.35	696.91	697.72	698.44
593-53-3	fluoromethane	CH_3F	C1s	296.32	296.90	297.41	296.73	297.31	297.82
75-46-7	trifluoromethane	CHF_3	F1s	698.57	699.37	700.14	698.63	699.52	700.38
75-46-7	trifluoromethane	CHF_3	C1s	301.27	301.89	302.50	301.97	302.66	303.33
67-56-1	methanol	CH_3OH	O1s	543.64	543.94	544.20	543.84	544.15	544.42
67-56-1	methanol	CH_3OH	C1s	295.39	295.85	296.24	295.74	296.24	296.67
50-00-0	formaldehyde	CH_2O	O1s	544.35	544.54	544.70	544.52	544.74	544.94
50-00-0	formaldehyde	CH_2O	C1s	297.24	297.77	298.25	297.70	298.24	298.73

Table S5: Continued

CAS	name	formula	core level	$G_{RS}W_{RS}$@PBE $n=3$	$G_{RS}W_{RS}$@PBE $n=4$	$G_{RS}W_{RS}$@PBE ∞	$G_{RS}W_{RS}$@PBE0 $n=3$	$G_{RS}W_{RS}$@PBE0 $n=4$	$G_{RS}W_{RS}$@PBE0 ∞
115-10-6	dimethyl ether	CH_3OCH_3	O1s	543.62	543.85	544.06	543.76	544.02	544.24
115-10-6	dimethyl ether	CH_3OCH_3	C1s	295.29	295.75	296.15	295.56	296.13	296.61
64-18-6	formic acid	HCOOH	O1s (OH)	545.48	545.71	545.93	545.70	545.95	546.19
64-18-6	formic acid	HCOOH	O1s (C=O)	543.88	544.09	544.27	544.12	544.32	544.52
64-18-6	formic acid	HCOOH	C1s	298.18	298.71	299.21	298.81	299.37	299.90
67-64-1	acetone	$(CH_3)_2CO$	O1s	542.78	543.34	543.83	542.92	543.59	544.19
67-64-1	acetone	$(CH_3)_2CO$	C1s (C=O)	296.63	297.09	297.50	297.10	297.62	298.08
67-64-1	acetone	$(CH_3)_2CO$	C1s (CH_3)	294.28	294.73	295.13	294.64	295.13	295.56
107-31-3	methyl formate	HCO_2CH_3	O1s (OCH_3)	545.12	545.26	545.39	545.32	545.51	545.68
107-31-3	methyl formate	HCO_2CH_3	O1s (C=O)	543.61	543.74	543.87	543.84	544.03	544.19
64-19-7	acetic acid	CH_3COOH	O1s (OH)	545.01	545.22	545.42	545.26	545.51	545.75
64-19-7	acetic acid	CH_3COOH	O1s (C=O)	543.28	543.49	543.69	543.48	543.77	544.03
64-19-7	acetic acid	CH_3COOH	C1s (COOH)	297.90	298.37	298.81	298.50	299.05	299.54
64-19-7	acetic acid	CH_3COOH	C1s (CH_3)	294.62	295.10	295.53	295.00	295.51	295.97
7732-18-5	water	H_2O	O1s	543.92	544.30	544.63	544.13	544.52	544.85
10028-15-6	ozone	O_3	O1s middle	551.82	551.98	552.15	552.50	552.69	552.88
10028-15-6	ozone	O_3	O1s terminal	546.85	547.00	547.15	547.36	547.51	547.65
7782-44-7	oxygen	O_2	O1s weaker						
7782-44-7	oxygen	O_2	O1s stronger						
7727-37-9	nitrogen	N_2	N1s	413.25	413.72	414.20	413.69	414.17	414.64
7664-41-7	ammonia	NH_3	N1s	409.21	409.76	410.22	409.50	410.06	410.54
74-90-8	hydrogen cyanide	HCN	N1s	410.79	411.13	411.45	411.16	411.53	411.88
74-90-8	hydrogen cyanide	HCN	C1s	296.04	296.52	296.98	296.49	296.94	297.36
75-05-8	acetonitrile	CH_3CN	N1s	409.55	410.15	410.68	409.80	410.58	411.29
75-05-8	acetonitrile	CH_3CN	C1s (CH_3)	295.68	296.14	296.56	296.13	296.54	296.91
75-05-8	acetonitrile	CH_3CN	C1s (CN)	295.44	295.87	296.25	295.89	296.32	296.70
56-40-6	glycine	$C_2H_5NO_2$	O1s (OH)	545.08	545.32	545.54	545.36	545.58	545.77
56-40-6	glycine	$C_2H_5NO_2$	O1s (C=O)	542.72	543.70	544.59	542.77	543.93	544.99
56-40-6	glycine	$C_2H_5NO_2$	N1s	409.43	409.94	410.41	409.76	410.27	410.74
56-40-6	glycine	$C_2H_5NO_2$	C1s (COOH)	297.69	298.27	298.81	298.34	298.93	299.46
56-40-6	glycine	$C_2H_5NO_2$	C1s (CH_2)	295.19	295.81	296.37	295.76	296.23	296.66
110-86-1	pyridine	C_5H_5N	N1s	409.27	409.71	410.12	409.63	410.12	410.58
109-97-7	pyrrole	C_4H_4NH	N1s	410.59	410.99	411.36	410.91	411.33	411.70
62-53-3	aniline	$C_6H_5NH_2$	N1s	409.66	410.05	410.40	409.93	410.35	410.73
57-13-6	urea	$CO(NH_2)_2$	O1s	542.32	542.47	542.60	542.55	542.73	542.89
57-13-6	urea	$CO(NH_2)_2$	N1s	410.13	410.56	410.95	410.45	410.94	411.39
57-13-6	urea	$CO(NH_2)_2$	C1s	297.31	297.86	298.36	297.98	298.57	299.10
74-89-5	methylamine	CH_3NH_2	N1s	409.07	409.58	410.01	409.36	409.87	410.31
98-95-3	nitrobenzene	$C_6H_5NO_2$	O1s	544.21	544.38	544.54	544.58	544.78	544.97
98-95-3	nitrobenzene	$C_6H_5NO_2$	N1s	415.63	416.07	416.48	416.46	416.98	417.47
98-95-3	nitrobenzene	$C_6H_5NO_2$	C1s (C1)	295.59	296.00	296.38	296.04	296.49	296.91
98-95-3	nitrobenzene	$C_6H_5NO_2$	C1s (C2-4)	294.76	295.13	295.48	295.18	295.59	295.98
71-43-2	benzene	C_6H_6	C1s	293.91	294.28	294.62	294.29	294.75	295.16
536-74-3	phenylacetylene	C_8H_6	C1s (C3)	294.58	294.99	295.37	295.00	295.43	295.83
536-74-3	phenylacetylene	C_8H_6	C1s (C2)	294.07	294.46	294.83	294.44	294.89	295.30
536-74-3	phenylacetylene	C_8H_6	C1s (C4-6)	294.07	294.46	294.81	294.48	294.90	295.29
536-74-3	phenylacetylene	C_8H_6	C1s (C1)	293.64	294.22	294.74	293.92	294.67	295.36

Table S6: Non-relativistic core-level binding energies from $G_{RS}W_{RS}$@B3LYP and $G_{RS}W_{RS}$@PBEh(α=0.45) using the cc-pVnZ basis set series with $n = 3, 4$ and 2-point extrapolated values (∞). For $G_{RS}W_{RS}$ calculations, the cc-pVnZ basis sets are treated as Cartesian Gaussian functions. All values in eV.

				$G_{RS}W_{RS}$@B3LYP			$G_{RS}W_{RS}$@PBEh(α=0.45)		
CAS	name	formula	core level	$n = 3$	$n = 4$	∞	$n = 3$	$n = 4$	∞
74-82-8	methane	CH_4	C1s	293.83	294.33	294.73	294.31	294.90	295.38
74-84-0	ethane	C_2H_6	C1s	293.89	294.31	294.66	294.39	294.92	295.36
74-85-1	ethene	C_2H_4	C1s	294.34	294.68	294.99	294.81	295.33	295.78
74-86-2	ethyne	C_2H_2	C1s	294.58	295.00	295.37	295.16	295.64	296.07
630-08-0	carbon monoxide	CO	O1s	546.81	546.96	547.11	547.37	547.60	547.83
630-08-0	carbon monoxide	CO	C1s	298.68	299.16	299.65	299.26	299.88	300.49
124-38-9	carbon dioxide	CO_2	O1s	545.98	546.11	546.23	546.65	546.91	547.16
124-38-9	carbon dioxide	CO_2	C1s	300.24	300.81	301.38	301.00	301.77	302.55
75-73-0	tetrafluoromethane	CF_4	F1s	699.38	699.96	700.53	699.84	700.44	701.04
75-73-0	tetrafluoromethane	CF_4	C1s	304.42	305.06	305.70	305.19	305.95	306.70
593-53-3	fluoromethane	CH_3F	F1s	696.56	697.32	698.00	696.91	697.79	698.57
593-53-3	fluoromethane	CH_3F	C1s	296.53	297.11	297.62	297.06	297.73	298.33
75-46-7	trifluoromethane	CHF_3	F1s	698.40	699.22	700.02	698.66	699.64	700.59
75-46-7	trifluoromethane	CHF_3	C1s	301.82	302.54	303.23	302.51	303.30	304.07
67-56-1	methanol	CH_3OH	O1s	543.35	543.65	543.91	543.98	544.36	544.70
67-56-1	methanol	CH_3OH	C1s	295.54	296.02	296.44	296.03	296.64	297.18
50-00-0	formaldehyde	CH_2O	O1s	544.06	544.26	544.45	544.64	544.92	545.16
50-00-0	formaldehyde	CH_2O	C1s	297.50	298.04	298.53	298.05	298.70	299.29
115-10-6	dimethyl ether	CH_3OCH_3	O1s	543.23	543.46	543.66	543.88	544.20	544.48
115-10-6	dimethyl ether	CH_3OCH_3	C1s	295.36	295.90	296.37	295.83	296.52	297.11
64-18-6	formic acid	HCOOH	O1s (OH)	545.23	545.47	545.69	545.88	546.20	546.50
64-18-6	formic acid	HCOOH	O1s (C=O)	543.67	543.86	544.03	544.29	544.55	544.79
64-18-6	formic acid	HCOOH	C1s	298.63	299.18	299.70	299.28	299.99	300.64
67-64-1	acetone	$(CH_3)_2CO$	O1s	542.52	543.11	543.63	543.02	543.81	544.51
67-64-1	acetone	$(CH_3)_2CO$	C1s (C=O)	296.83	297.35	297.81	297.51	298.13	298.68
67-64-1	acetone	$(CH_3)_2CO$	C1s (CH_3)	294.38	294.88	295.33	294.92	295.50	296.02
107-31-3	methyl formate	HCO_2CH_3	O1s (OCH_3)	544.81	544.96	545.10	545.47	545.72	545.96
107-31-3	methyl formate	HCO_2CH_3	O1s (C=O)	543.38	543.54	543.68	544.01	544.26	544.49
64-19-7	acetic acid	CH_3COOH	O1s (OH)	544.79	545.02	545.24	545.45	545.78	546.08
64-19-7	acetic acid	CH_3COOH	O1s (C=O)	543.06	543.30	543.51	543.63	544.00	544.34
64-19-7	acetic acid	CH_3COOH	C1s (COOH)	298.26	298.81	299.32	298.98	299.68	300.32
64-19-7	acetic acid	CH_3COOH	C1s (CH_3)	294.75	295.25	295.71	295.29	295.89	296.43
7732-18-5	water	H_2O	O1s	543.70	544.09	544.43	544.30	544.73	545.11
10028-15-6	ozone	O_3	O1s middle	551.95	552.12	552.28	553.03	553.39	553.75
10028-15-6	ozone	O_3	O1s terminal	546.93	547.06	547.19	547.72	547.96	548.21
7782-44-7	oxygen	O_2	O1s weaker						
7782-44-7	oxygen	O_2	O1s stronger						
7727-37-9	nitrogen	N_2	N1s	413.34	413.80	414.27	414.03	414.60	415.18
7664-41-7	ammonia	NH_3	N1s	409.11	409.67	410.15	409.73	410.37	410.91
74-90-8	hydrogen cyanide	HCN	N1s	410.75	411.11	411.45	411.44	411.91	412.35
74-90-8	hydrogen cyanide	HCN	C1s	296.05	296.70	297.31	296.88	297.35	297.80
75-05-8	acetonitrile	CH_3CN	N1s	409.56	410.15	410.68	410.59	410.98	411.34
75-05-8	acetonitrile	CH_3CN	C1s (CH_3)	295.78	296.27	296.72	296.40	296.93	297.42
75-05-8	acetonitrile	CH_3CN	C1s (CN)	295.61	296.03	296.41	296.23	296.82	297.36
56-40-6	glycine	$C_2H_5NO_2$	O1s (OH)	544.88	545.09	545.28	545.56	545.85	546.12
56-40-6	glycine	$C_2H_5NO_2$	O1s (C=O)	542.60	543.46	544.24	544.92	544.17	543.49
56-40-6	glycine	$C_2H_5NO_2$	N1s	409.30	409.81	410.28	410.00	410.60	411.14
56-40-6	glycine	$C_2H_5NO_2$	C1s (COOH)	298.12	298.69	299.21	298.87	299.55	300.17
56-40-6	glycine	$C_2H_5NO_2$	C1s (CH_2)	295.32	295.96	296.55	295.99	296.65	297.26

Table S6: Continued

CAS	name	formula	core level	$G_{RS}W_{RS}$@B3LYP			$G_{RS}W_{RS}$@PBE(α=0.45)		
				n = 3	n = 4	∞	n = 3	n = 4	∞
110-86-1	pyridine	C_5H_5N	N1s	409.14	409.61	410.05	409.90	410.51	411.07
109-97-7	pyrrole	C_4H_4NH	N1s	410.41	410.82	411.19	411.15	411.69	412.18
62-53-3	aniline	$C_6H_5NH_2$	N1s	409.48	409.89	410.26	410.15	410.59	410.99
57-13-6	urea	$CO(NH_2)_2$	O1s	542.13	542.26	542.37	542.72	542.96	543.17
57-13-6	urea	$CO(NH_2)_2$	N1s	409.95	410.51	411.01	410.72	411.29	411.81
57-13-6	urea	$CO(NH_2)_2$	C1s	297.77	298.35	298.89	298.50	299.25	299.93
74-89-5	methylamine	CH_3NH_2	N1s	408.92	409.43	409.87	409.59	410.18	410.69
98-95-3	nitrobenzene	$C_6H_5NO_2$	O1s	544.10	544.26	544.42	544.82	545.05	545.26
98-95-3	nitrobenzene	$C_6H_5NO_2$	N1s	415.91	416.42	416.90	417.06	417.62	418.15
98-95-3	nitrobenzene	$C_6H_5NO_2$	C1s (C1)	295.73	296.14	296.53	296.41	296.88	297.33
98-95-3	nitrobenzene	$C_6H_5NO_2$	C1s (C2-4)	294.88	295.27	295.63	295.53	295.97	296.39
71-43-2	benzene	C_6H_6	C1s	293.97	294.37	294.72	294.58	295.03	295.44
536-74-3	phenylacetylene	C_8H_6	C1s (C3)	294.64	295.07	295.46	295.30	295.76	296.18
536-74-3	phenylacetylene	C_8H_6	C1s (C2)	294.14	294.56	294.95	294.77	295.22	295.63
536-74-3	phenylacetylene	C_8H_6	C1s (C4-6)	294.15	294.56	294.93	294.79	295.24	295.66
536-74-3	phenylacetylene	C_8H_6	C1s (C1)	293.72	294.31	294.86	294.58	295.00	295.39

5 ETFA molecule

Table S7: Core-level binding energies from different *GW* methods for the ETFA molecule using the cc-pV*n*Z basis set series with n = 3 – 6. Extrapolated values (∞), extrapolated values with relativistic correction (rel. corrected), standard error (SE) and correlation coefficient R^2. The last column indicates the basis sets used for the extrapolation. All values in eV.

method	core level	n = 3	n = 4	n = 5	n = 6	∞	rel. corrected	SE	R^2	n used
G_0W_0@PBEh	C1s (C1)	298.73	299.03	299.12	299.29	299.37	299.49	0.05	0.97	3-6
	C1s (C2)	295.64	295.91	295.99	296.15	296.23	296.34	0.05	0.97	3-6
	C1s (C3)	292.82	293.06	293.14	293.31	293.37	293.49	0.05	0.95	3-6
	C1s (C4)	290.97	291.20	291.29	291.45	291.51	291.63	0.05	0.95	3-6
$G_{\Delta H}W_0$@PBE	C1s (C1)	297.38	297.81	297.94	298.15	298.28	298.40	0.05	0.98	3-6
	C1s (C2)	294.42	294.77	294.93	295.14	295.24	295.36	0.07	0.97	3-6
	C1s (C3)	292.18	292.52	292.64	292.90	292.98	293.09	0.08	0.95	3-6
	C1s (C4)	290.58	290.91	291.01	291.26	291.33	291.45	0.08	0.94	3-6
evGW_0@PBE	C1s (C1)	297.20	297.62	297.77	297.99	298.11	298.23	0.06	0.98	3-6
	C1s (C2)	294.32	294.68	294.82	295.05	295.14	295.26	0.07	0.96	3-6
	C1s (C3)	292.08	292.41	292.57	292.79	292.88	293.00	0.07	0.96	3-6
	C1s (C4)	290.46	290.76	290.91	291.17	291.23	291.35	0.09	0.93	3-6
evGW@PBE	C1s (C1)	298.99	299.38	299.49	*	299.74	299.86	0.05	0.99	3-5
	C1s (C2)	296.00	296.36	296.47	*	296.70	296.81	0.04	0.99	3-5
	C1s (C3)	293.83	294.11	294.23	*	294.42	294.54	0.01	1.00	3-5
	C1s (C4)	292.20	292.48	292.57	*	292.75	292.87	0.03	0.99	3-5
$G_{RS}W_{RS}$@PBE	C1s (C1)	301.24	301.83	–	–	302.37	302.49	–	–	3-4
	C1s (C2)	298.38	298.90	–	–	299.38	299.50	–	–	3-4
	C1s (C3)	296.28	296.76	–	–	297.20	297.32	–	–	3-4
	C1s (C4)	294.64	295.08	–	–	295.49	295.61	–	–	3-4
$G_{RS}W_{RS}$@PBE0	C1s (C1)	302.06	302.66	–	–	303.22	303.34	–	–	3-4
	C1s (C2)	299.12	299.68	–	–	300.21	300.33	–	–	3-4
	C1s (C3)	296.69	297.22	–	–	297.72	297.84	–	–	3-4
	C1s (C4)	294.94	295.42	–	–	295.86	295.98	–	–	3-4
$G_{RS}W_{RS}$@B3LYP	C1s (C1)	301.85	302.45	–	–	303.01	303.13	–	–	3-4

Table S7: Continued

method	core level	n = 3	n = 4	n = 5	n = 6	∞	rel. corr.	SE	R^2	n used
	C1s (C2)	298.91	299.45	–	–	299.97	300.09	–	–	3-4
	C1s (C3)	296.44	296.94	–	–	297.40	297.52	–	–	3-4
	C1s (C4)	294.70	295.16	–	–	295.58	295.70	–	–	3-4
$G_{RS}W_{RS}$@PBEh(α=0.45)	C1s (C1)	302.68	303.27	–	–	303.83	303.95	–	–	3-4
	C1s (C2)	299.65	300.25	–	–	300.81	300.93	–	–	3-4
	C1s (C3)	297.01	297.57	–	–	298.09	298.21	–	–	3-4
	C1s (C4)	295.20	295.70	–	–	296.17	296.29	–	–	3-4

6 Graphical solution from $G_{RS}W_0$@B3LYP for O1s in H_2O

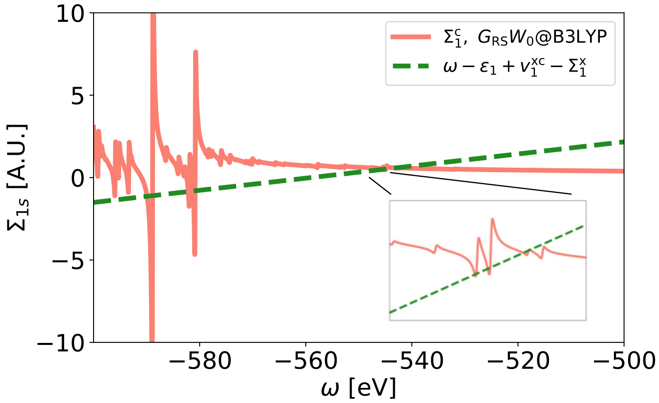

Figure S1: The graphical solution of O1s excitation for a single water molecule obtained from $\boldsymbol{G_{RS}W_0}$@B3LYP, the cc-pVQZ basis set was used.

7 Errors of CLBEs and ΔCLBEs obtained from KS-DFT calculations

Table S8: MAEs and MEs in eV with respect to experiment for CLBEs of the CORE65 benchmark set obtained from KS-DFT eigenvalues. Calculations were performed with the QM4D package.

	PBE		PBE0		B3LYP		PBEh	
	MAE	ME	MAE	ME	MAE	ME	MAE	ME
C1s	22.15	-22.15	12.80	-12.80	14.10	-14.10	2.56	-2.56
N1s	24.88	-24.88	13.95	-13.95	15.72	-15.72	1.79	-1.79
O1s	27.96	-27.96	15.47	-15.47	17.57	-17.57	1.01	-0.94
F1s	32.39	-32.39	18.35	-18.35	20.72	-20.72	1.62	-1.62

* evGW outer cycle not converged

Table S9: MAEs and MEs in eV with respect to experiment for ΔCLBEs of the CORE65 benchmark set obtained from KS-DFT eigenvalues. The ΔCLBEs are the shifts with respect to a reference molecule, ΔCLBE = CLBE − $CLBE_{ref\_mol}$. CH_4, NH_3, H_2O and CH_3F have been used as reference molecules for C1s, N1s, O1s and F1s respectively. Calculations were performed with the QM4D package.

	PBE		PBE0		B3LYP		PBEh	
	MAE	ME	MAE	ME	MAE	ME	MAE	ME
C1s	0.69	0.13	0.64	0.28	0.60	0.26	0.72	-0.22
N1s	0.68	0.67	0.75	0.73	0.68	0.66	1.16	1.12
O1s	1.00	1.16	1.06	1.14	0.97	1.07	1.28	1.26
F1s	0.23	0.23	0.16	0.16	0.15	0.15	0.13	0.13

8 Starting point dependence

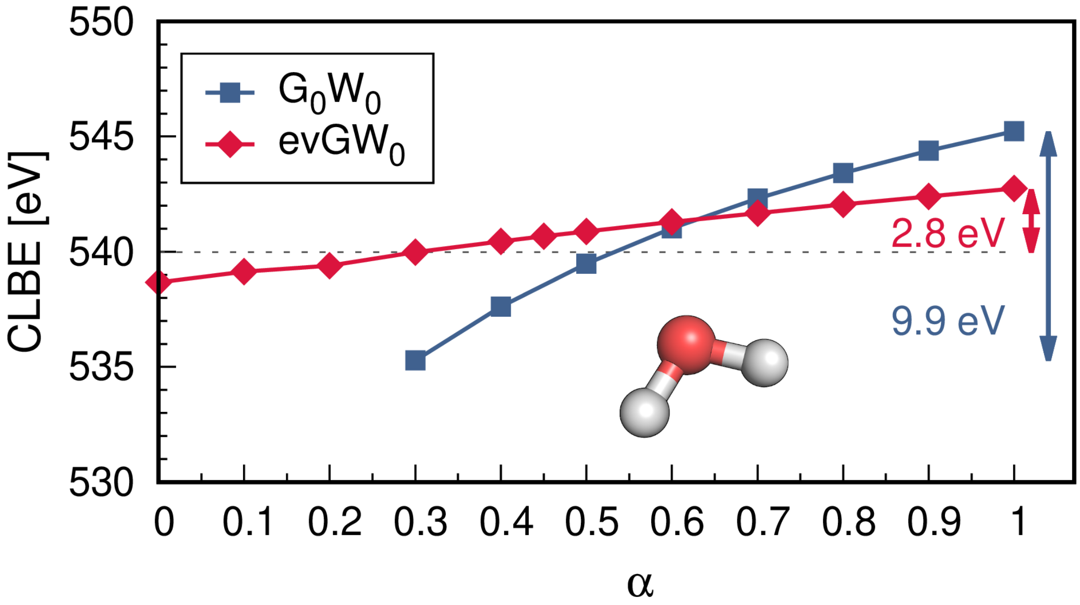

Figure S2: Dependence of the O1s CLBE of H_2O on the amount of exact exchange α with G_0W_0@PBEh(α) and evGW_0@PBEh(α). The CLBEs are obtained at the cc-pV4Z level. Relativistic corrections are not included.

9 ETFA geometry in xyz format

```
14

 C      1.74342172      0.79233009     -2.38607740
 C      1.87225299      0.82531137     -0.83161745
 C      1.85983154     -0.55069881      1.10298970
 C      1.77609805     -2.02516635      1.42902523
 F      1.98731130      2.01031027     -2.90139852
 F      2.61557025     -0.08958676     -2.93832354
 F      0.48395202      0.42008648     -2.74324352
 O      2.00651584      1.84892786     -0.20216186
 O      1.78925481     -0.42316165     -0.35803629
 H      2.80240352     -0.09477382      1.43702621
 H      1.02799211      0.02628227      1.53157701
 H      0.83503378     -2.45818712      1.06497140
 H      2.61261424     -2.57849042      0.98265727
 H      1.81641784     -2.15921342      2.51833177
```

References

(1) Golze, D.; Keller, L.; Rinke, P. Accurate Absolute and Relative Core-Level Binding Energies from *GW*. *J. Phys. Chem. Lett* **2020**, *11*, 1840–1847.

(2) Keller, L.; Blum, V.; Rinke, P.; Golze, D. Relativistic correction scheme for core-level binding energies from *GW*. *J. Chem. Phys.* **2020**, *153*, 114110.
